# The Marcus dimension: identifying the nuclear coordinate for electron transfer from *ab initio* calculations†

**DOI:** 10.1039/d3sc01402a

**Published:** 2023-08-08

**Authors:** Adam Šrut, Benjamin J. Lear, Vera Krewald

**Affiliations:** a Department of Chemistry, Theoretical Chemistry, TU Darmstadt Peter-Grünberg-Straße 4 64287 Darmstadt Germany vera.krewald@tu-darmstadt.de; b Department of Chemistry, The Pennsylvania State University University Park PA 16802 USA bul14@psu.edu

## Abstract

The Marcus model forms the foundation for all modern discussion of electron transfer (ET). In this model, ET results in a change in diabatic potential energy surfaces, separated along an ET nuclear coordinate. This coordinate accounts for all nuclear motion that promotes electron transfer. It is usually assumed to be dominated by a collective asymmetric vibrational motion of the redox sites involved in the ET. However, this coordinate is rarely quantitatively specified. Instead, it remains a nebulous concept, rather than a tool for gaining true insight into the ET pathway. Herein, we describe an *ab initio* approach for quantifying the ET coordinate and demonstrate it for a series of dinitroradical anions. Using sampling methods at finite temperature combined with density functional theory calculations, we find that the electron transfer can be followed using the energy separation between potential energy surfaces and the extent of electron localization. The precise nuclear motion that leads to electron transfer is then obtained as a linear combination of normal modes. Once the coordinate is identified, we find that evolution along it results in a change in diabatic state and optical excitation energy, as predicted by the Marcus model. Thus, we conclude that a single dimension of the electron transfer described in Marcus–Hush theory can be described as a well-defined nuclear motion. Importantly, our approach allows the separation of the intrinsic electron transfer coordinate from other structural relaxations and environmental influences. Furthermore, the barrier separating the adiabatic minima was found to be sufficiently thin to enable heavy-atom tunneling in the ET process.

## Abstract 2

The electron transfer coordinate of the Marcus–Hush model is recovered from *ab initio* calculations.

## Graphical abstract

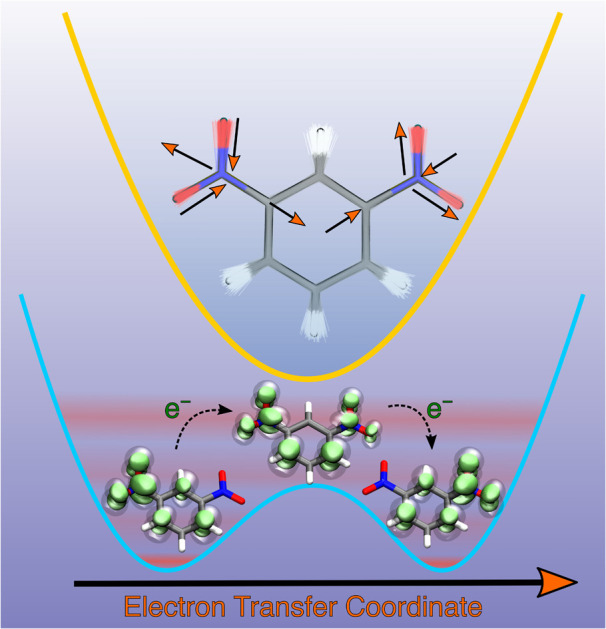

## 1 Introduction

The transfer of electron density is implicated in nearly every chemical transformation and hence there has been a long-standing and intense interest in developing models for describing, predicting, and quantifying the pathways of electron transfer (ET). For the past 60 years, the dominant model has been the classical Marcus–Hush theory.^1,2^

**Fig. 1:**
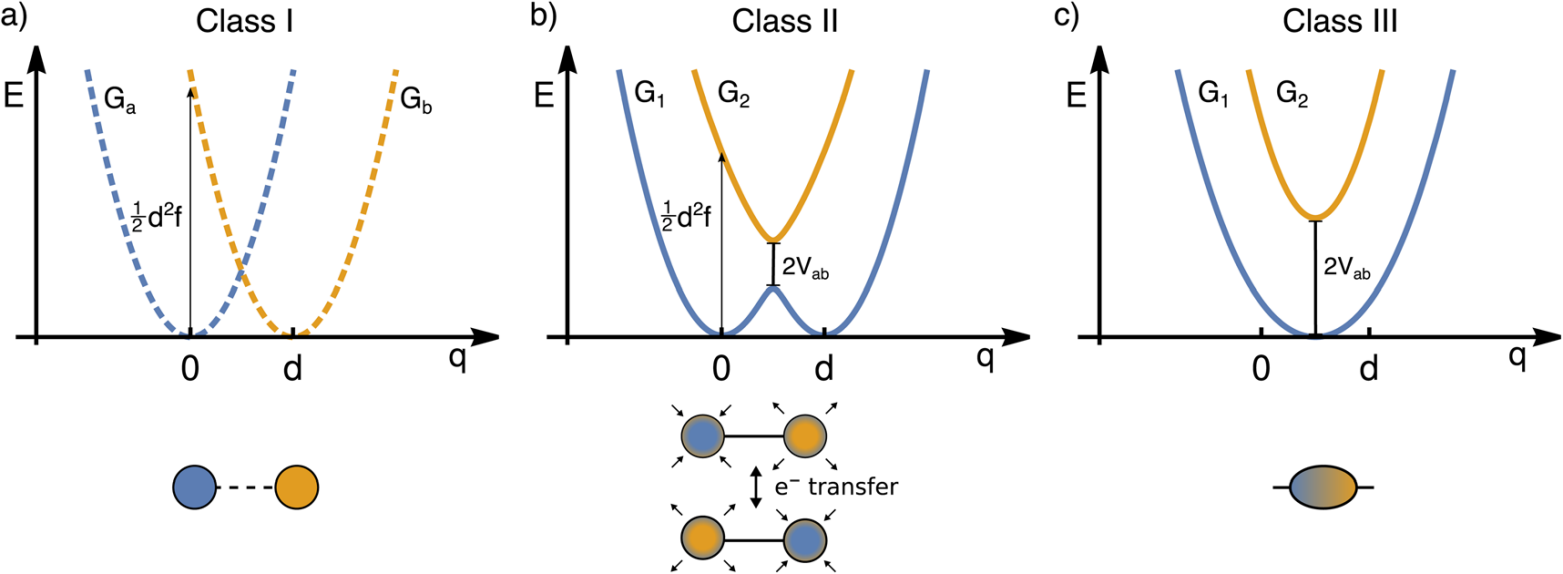
One dimensional potential curves of Robin–Day classes ((a) class I, (b) class II, (c) class III) separated along the ET coordinate (*q*). The vertical arrow of length 
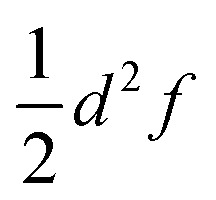
 represents the reorganization energy. *V*_ab_ is the potential coupling between the initial diabatic states G_a_ and G_b_ that leads to the adiabatic states G_1_ and G_2_.

The theory originally described by Marcus treats ET between two separated redox sites (*i.e.*, intermolecular ET), giving rise to two electronic states a and b. These states are represented on a reaction coordinate diagram by two harmonic potential energy surfaces (PES) as shown in Fig. 1a. The electron transfer occurs when the nuclei (internal and solvent) are distorted such that the two surfaces have the same energy, at which point there is some probability of ET. This can be represented using Fermi's golden rule^3,4^1
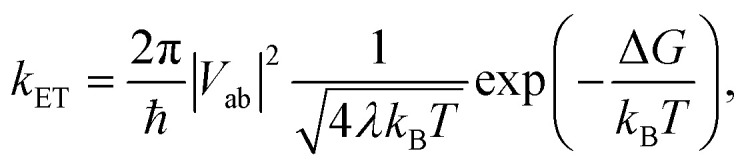
where *V*_ab_ is the electronic coupling between the states (taken to be small in the original treatment), Δ*G* is the free energy of activation, and *λ* is the so-called reorganization energy, which is the vertical energy gap between the reactant minima and the product PES. The reorganization energy can be obtained by measuring this electronic transition, termed the intervalence charge transfer (IVCT) transition. Because the surfaces are treated as harmonic surfaces, measuring the IVCT energy provides the barrier to thermal ET: *λ*/4. Though this is a convenient way to parameterize the model, it disregards the specifics of the ET coordinate. A more chemically meaningful treatment would quantify the nuclear motions involved in the ET. This would allow to derive a spring constant (*f*) for this motion as well as the separation of the minima (*d*) of the PES along the ET coordinate so that the reorganization energy could be recaptured as 
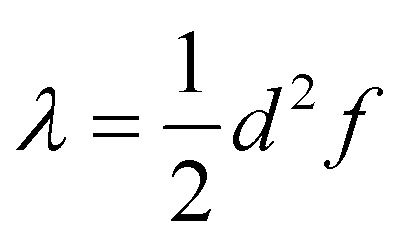
.

The insight that would be gained by obtaining a precise description of the ET coordinate is made clear when considering systems with large degrees of electronic coupling. Hush expanded Marcus' theory for such systems, retaining the connection between the electron transfer coordinate, the barrier to electron transfer, the curvature of the PES, and the displacement along the ET coordinate. Again, it was common to combine the effects of curvature and separation into a reorganization energy, though some treatments explicitly considered the spring constant and separation.^5^

Large electronic coupling mixes the diabatic states G_a_ and G_b_ and produces two adiabatic states: a ground state G_1_ and an excited state G_2_ (Fig. 1b and c). Thus, for significant coupling, ET will occur adiabatically, in which case the pre-exponential factor will reduce to a nuclear frequency along the ET coordinate *ν*_*q*_. The resulting classical expression for the ET rate will be:^6^2
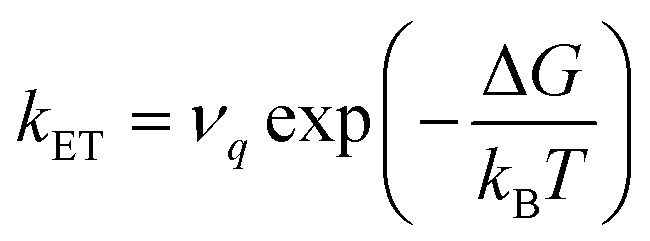
where correct prediction of *ν*_*q*_ requires identifying the nuclear motions involved in ET.

The ET coordinate is also implicated in other effects of electronic coupling, namely that increasing electronic coupling produces a movement of the ground state PES minima towards one another along the ET coordinate. If the coupling is strong enough, the minima merge, and the molecule has a single stable configuration with the unpaired electron delocalized over the redox sites. The shape of the ground state PES is the basis for the dominant classification scheme for mixed valence systems proposed by Robin and Day.^7^ In this classification, systems with minimal coupling (*i.e.*, diabatic) are termed Class I, systems with a single minimum are termed Class III, and the intermediate case is termed Class II. Movement along *q* away from the minimum in Class III therefore implies a greater extent of localisation, whereas movement along *q* between the minima in Class II leads to electron transfer.

While electronic coupling in these complexes is often discussed without reference to the PES spring constant and minima separation, it is also true that the final shape of the surface depends not only on the magnitude of the electronic coupling, but also on the curvature and separation along the ET coordinate.^8^ It is well established that solvation has a pronounced influence on the shape of the potential and can even lead to a switch in Class.^9,10^ Having a direct way of identifying the shape of the PES would therefore be of great value for disentangling these different contributors, comparing the properties of different chemical systems in more detail, and quantifying environmental effects.

The lack of attention paid to the spring constant and separation between PES minima comes from both experimental and theoretical limitations. Experimentally, the IVCT band can be used to estimate the magnitude of electronic coupling,^9,11^ although it contains limited information about the spring constant and the separation of the PES minima. Vibronic progressions and resonance Raman experiments can shed some light on these, although some contributing motions may not be captured. The donor–acceptor distance, which is not identical to the separation of the adiabatic minima on the nuclear coordinate *q*, can be evaluated from the change in dipole moment upon IVCT excitation.^12,13^ Deducing the electronic coupling from changes in the dipole moment is hampered by the fact that estimating the change in dipole moment in the absence of coupling is difficult.^14,15^ Finally, even if the approach were valid, what would be measured is not the ET coordinate directly, but a separation along it. Therefore, any specific chemical information about the nuclear motions involved is not obtained.

On the theory side, one can perform a linear interpolation between the reactants and transition state, or the reactants and the products, as is common in light-induced ET to understand which nuclear motions are implicated in ET. However, this approach is also restricted to Class II systems where two well defined minima and a transition state exist. For MV systems that are sensitive to the solvation environment, a linear interpolation approach would not be able to evaluate any solvent-induced change in nuclear motion or the decoupling of solvent and solute motion. Finally, as shown below, a linear interpolation approach may furthermore contain motions that are not required for the ET process and thus render a coordinate with superfluous motions.

Considering the state of the art in ET research, there remains a need for a general method to describe the ET coordinate quantitatively. Ideally such a method would capture details such as the nature of nuclear motion promoting ET, the spring constant associated with this motion, and the separation of minima along potential energy surfaces associated with this coordinate. Additionally, it would be easily expandable to include additional dimensions of electron transfer, *e.g.* to evaluate the relevance of higher-lying excited states. Herein, we describe one such approach based upon quantum chemical calculations, demonstrating it for organic mixed valence systems.

The method we propose can identify the ET coordinate of a MV system regardless of its Robin–Day Class, which is achieved with a sampling procedure covering a representative set of thermally populated nuclear configurations and subsequent analysis of the electronic structures in this ensemble. The coordinate we identify appears to be an intrinsic property of the mixed valent molecule and can be used to predict the barrier height or reorganization energy of the system. We demonstrate that this intrinsic motion is separable from structural relaxations that do not contribute to driving ET and from environmental influences. We also show that, while this approach largely substantiates the Marcus–Hush model, it also raises some questions regarding interpretation of the model and reveals some of its limitations.

## 2 Methodology

For many years, computational predictions of spectroscopic properties of MV compounds were hampered by the fact that most theoretical methods tend to favour either a localized or a delocalized description. Solutions to this problem were found and discussed in seminal work by Martin Kaupp and coworkers.^16^ Herein, we use local hybrid functionals that are designed to strike a balance between correcting the self-interaction error and an accurate description of exchange–correlation^17,18^ and have been shown to perform extraordinarily well in the prediction of spectroscopic properties of MV compounds.^19–21^

Theoretical studies on MV systems often assume a direct pathway between two well-defined structures, either the adiabatic minima^22^ or an adiabatic minimum and a totally symmetric structure.^23^ Powerful approaches to recover the potential energy curves from Marcus theory using the energy gap between the diabatic states have been established.^24,25^ To identify the diabatic states, one can either use constrained DFT,^26,27^ where they result directly from the calculation, or use a diabatization procedure.^28^ However, both of these approaches will be challenging for strongly coupled systems and will not provide information about the curvature of the potential energy curves.

To assess the interplay between the geometric and electronic structure of a MV system, it is clear that the picture derived from a single nuclear configuration will not represent reality. Therefore, a sampling procedure is needed that covers a set of thermally populated nuclear configurations with their associated electronic structures. The nuclear ensemble method^29^ presents a simple and powerful method for simulating vibrationally resolved electronic spectra,^30,31^ for obtaining the initial conditions for non-adiabatic dynamics,^32^ or for exploring phase space properties.^33^ The most common way to generate a nuclear ensemble is *ab initio* molecular dynamics which, however, comes with a great computational cost.^34^

A significantly less demanding method for moderately sized molecules is the so-called Wigner sampling,^35^ which is achieved by approximating the PES by a harmonic potential and evaluating the Wigner function^36^ for thermally accessible vibrational states. The Wigner sampling method showed great performance when predicting absorption spectra^37^ and capturing temperature effects in the intersystem crossing of 2-nitronaphthalene.^38^ A strength of Wigner sampling is that it offers a more realistic sampling than *ab initio* molecular dynamics for high-frequency vibrational modes, because it accounts for the zero-point energy.^37^ However, it does not capture the decreasing spacing between levels and altered nature that is expected for an anharmonic oscillator and so undersamples anharmonic low-frequency modes. While we use Wigner sampling here, it is conceivable that other sampling methods will perform equally well as long as a representative set of geometric and electronic structures is used.

**Fig. 2:**
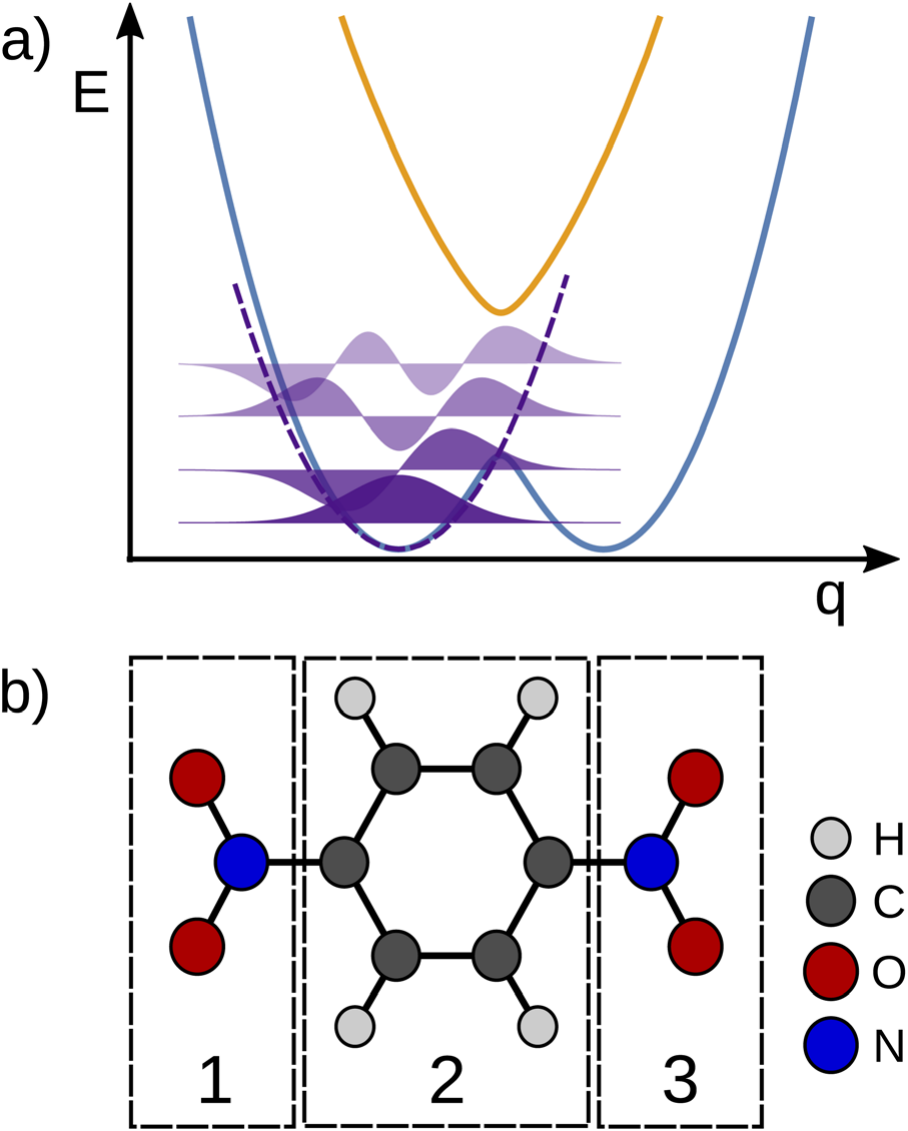
(a) Schematic depiction of the approximated potential energy surface (black curve) employed in the Wigner sampling method. (b) Definition of chemical fragments in a MV molecule to identify the electron position from a spin population analysis.

To the best of our knowledge, a unified strategy towards a coordinate that describes electron transfer (Class II) or localisation (Class III) and that can be used to disentangle intra-molecular motion from solvent motion is missing in state-of-the-art theoretical research of MV systems. Our approach for identifying the intramolecular ET coordinate starts from an optimized geometry that represents an adiabatic minimum of the system and the Hessian matrix obtained from a frequency calculation. The phase space of the system under study is sampled using the Wigner sampling method as implemented in the SHARC package^39^ at room temperature. The system can be placed in vacuum or in implicit solvation; while the inclusion of explicit solvation is not discussed here this should be equally feasible with the Wigner method or other sampling approaches.^37^ The central sampling idea is illustrated in Fig. 2a where the potential energy surface is approximated around the adiabatic minimum by a harmonic potential. A representative set of nuclear configurations is selected by evaluating the Wigner function for the relevant vibrational states whose thermal populations are estimated in Monte Carlo fashion.^39^ The next step is to run time-dependent DFT (TD-DFT) calculations with a small number of excited states for each selected geometry.

For each of the structures, it can be easily evaluated where the unpaired electron is localized: the position of this electron is defined as the weighted average of the spin populations *s*_*i*_ on specified molecular fragments, as illustrated in Fig. 2b and given in eqn (3). The electron position is a unitless scalar number that takes value between 1 and 3, noting that the choice of integers for the fragments is up to the user. The quantity can be thought of as a centroid of the spin density.

3 e_pos._^−^ = 1 × *s*_1_ + 2 × *s*_2_ + 3 × *s*_3_.

To identify the ET coordinate, the electronic properties, *i.e.*, the electron position or the excitation energy as discussed in more detail in the results section, are correlated with the distortion in the direction of vibrational modes. To obtain these displacements, a transformation from Cartesian coordinates to normal coordinates is carried out according to eqn (4), where **C** is a transformation matrix (obtained from the frequency calculation at the adiabatic minimum), **M** is a diagonal matrix of atomic masses, **r** is a vector of Cartesian coordinates, and **r**^0^ is the equilibrium configuration,4
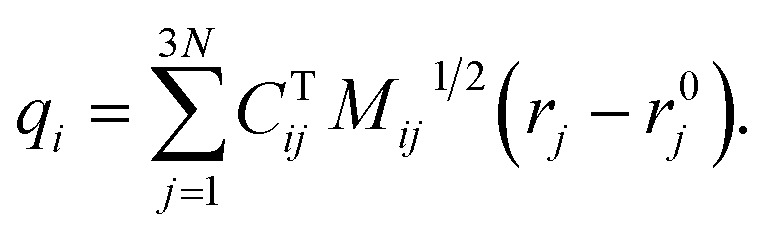


## 3 Choice of model systems

**Fig. 3:**
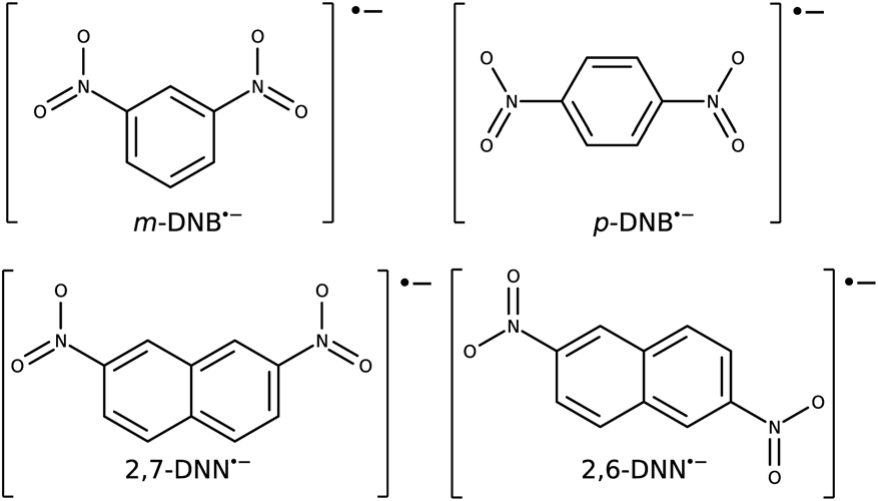
Structures and charge states of the studied mixed valence systems for which we demonstrate our approach.

As test cases, we have chosen *meta*-dinitrobenzene (*m*-DNB˙^−^), *para*-dinitrobenzene (*p*-DNB˙^−^), 2,6-dinitronaphthalene (2,6-DNN˙^−^), and 2,7-dinitronaphthalene (2,7-DNN˙^−^), see Fig. 3. These molecules have been well characterized experimentally and computationally so that the IVCT bands and ET rates are known. They served as model compounds for exploring the adiabatic ET rate^40^ or for development of reliable electronic structure methods for MV systems.^23^ These extensive studies render the four dinitrocompounds as ideal test cases for our proof-of-concept study.

The Robin–Day classification of these compounds depends on the solvation environment.^41^ In vacuum, all four compounds are expected to be Class III, while when placed in a dielectric continuum of acetonitrile, *m*-DNB˙^−^ and 2,7-DNN˙^−^ transition to Class II.^16^ Alcohol solvents, which are capable of hydrogen bonding, show a very strong localization effect and high ET barriers.^41^

## 4 Computational details

All calculations were carried out using the TURBOMOLE package.^42^ The local hybrid functional^43–45^ LH20t^19^ was employed; in the ESI† we show additional tests using the BLYP35 (ref. 46) functional. Calculations used the def2-TZVP basis set^47^ for the carbon, nitrogen and oxygen atoms, and the def2-SVP basis set^47^ for hydrogen atoms. The resolution of identity approximation for computation of the Coulomb integrals^48^ was used. The convergence criterion for the self-consistent field method was set to 10^−8^*E*_h_. As the integration grid, m3 in TURBOMOLE notation was used to obtain the energy. Solvation effects were modelled implicitly using a conductor-like screening model^49^ (COSMO) with acetonitrile (ACN) as the modeled solvent. The five lowest excited states were determined using TD-DFT without the Tamm–Dancoff approximation and the same settings as described above. The phase space of all studied molecules was sampled using the Wigner sampling method as implemented in the SHARC package^39^ at 300 K. 500 structures were generated as representative of the ensemble. Any scans performed along linear interpolation coordinates or the electron transfer coordinates identified with our approach used the LH20t/def2-TZVP electronic structure method described above and a step size of 0.002 or 0.005 Å. To evaluate the electronic structure progression along the Marcus dimension, CASSCF and CASSCF/NEVPT2 calculations were performed using the ORCA suite of programs;^50^ the respective computational details are given in the ESI.†

To summarise, the calculations were carried out in the following order: (I) geometry optimization of the ground state and frequency calculation with DFT in vacuum or considering solvation effects implicitly if desired.‡‡As stated elsewhere in the text, the concept is expected to be easily translatable to explicit solvation environments using QM-MD, QM/MM-MD or other approaches for sampling a representative ensemble *via* snapshots. (II) Wigner sampling of 500 geometries at 300 K. (III) Single-point calculations of each sampled geometry with TD-DFT. (IV) Fitting of normal coordinates to the electron position or the excitation energy. (V) Linear combination of normal modes using the coefficients obtained from (IV) into the Marcus dimension. (VI) Scan along the Marcus dimension with TD-DFT.

## 5 Results and discussion

### 5.1 Electron transfer driven by vibrational modes

The ET coordinate represents nuclear movements associated with the transfer of the unpaired electron between the redox centres in the mixed-valent system. An intuitive concept of the ET coordinate is an anti-symmetric vibrational mode.^51–53^ In organic MV systems, the unpaired electron is often found on a multi-atom functional group, *i.e.* more delocalised than in inorganic complexes where the redox sites are mostly restricted to metal ions. Therefore, the ET coordinate cannot be conceived of as intuitively as in many inorganic complexes.^54^ Herein, we present a method for obtaining the ET coordinate from *ab initio* calculations representing an ensemble.

Our task is to find an association between the distortions in the direction of each vibrational mode with a property that is very sensitive to progression along the ET coordinate. Considering the Marcus–Hush model and the Robin–Day classification, appropriate electronic properties are the electron position in Class III cases, and the excitation energy in Class II cases. In Class III systems, even a small movement along the ET coordinate away from the adiabatic minimum can lead to a non-negligible degree of electron localisation on one of the redox centers. The electron position is thus an intuitive property to be correlated with vibrational motions. This is, however, not the case in Class II systems, where in the vicinity of the adiabatic minimum the unpaired electron stays localized on the respective redox center. A significant change of the electron position will occur only near the top of the barrier.

Instead, the property that can uniquely define the ET coordinate in Class II systems is the excitation energy to the first excited state. As the system progresses from the adiabatic minimum to the top of the barrier, the excitation energy will decrease. The nuclear configuration with the lowest excitation energy will thus correspond to the top of the barrier, as can be readily seen from the Marcus model (Fig. 1). We note that this approach is conceptually similar to following the energy gap between the diabatic states to obtain the ET coordinate.^9,25^ Class III systems cannot be treated this way because the excitation energy increases with the absolute value of nuclear displacements along the ET coordinate. Consequently, the information whether a negative or positive displacement of any vibrational mode pushes ET forwards or backwards, *i.e.* to the acceptor or donor, is lost. The calculated ground state electronic structure of the mixed valence state can be used to assign the molecule as Class II or III, thereby indicating which approach is appropriate.

**Fig. 4:**
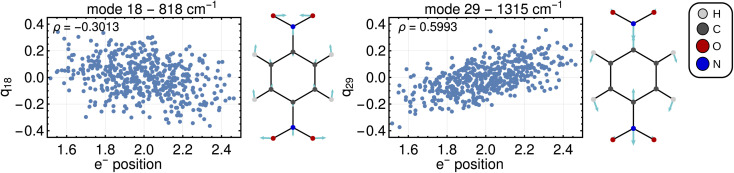
Normal coordinates plotted against the electron position with the corresponding vibrational mode for *p*-DNB˙^−^ in ACN. Plot labels show the number of the vibrational mode and its harmonic frequency, *ρ* in the top-left corner indicates the respective correlation coefficient. All other normal modes exhibit a lower absolute value of *ρ*.

Computing the correlation between a normal coordinate and the relevant ET property will reveal which vibrational modes contribute to the unique ET dimension. To this end, we plotted the relevant ET properties against the normal coordinates, see Fig. 4 for a Class III case and Fig. S2† for a Class II case. We focus here on the Class III case because we are not aware of alternative methods to gain insight into the ET coordinate for Class III systems. In the Class III example shown in Fig. 4, a correlation with the electron position can be seen for some normal coordinates. The two most strongly pronounced correlations are those with the antisymmetric stretching of C–N bonds (mode 29) which is apparent at first glance, and the correlation with the ONO bending motion (mode 18) that is less clear. The importance of these modes for the ET dimension agrees with chemical expectations, *i.e.*, the ET is associated with changing the distance between redox center (NO_2_ group) and bridging unit (benzene ring). Very similar observations are made for a Class II system where, as explained above, the correlation is established using the excitation energy (see Fig. S2†).

In both cases, there is no ultimate measure for deciding whether or how much these modes contribute to the overall ET dimension. In other words, it is not clear how much they contribute to the reorganization energy *λ*. Correlating the normal coordinate with the relevant ET property can only identify the relative importance of the vibrational modes. This finding underscores the need for a unified ET coordinate for which one would then know the spring constant *f* and the separation of the minima *d*, and therefore *λ*.

### 5.2 Marcus dimension of the electron transfer

As shown above, the ET coordinate cannot be assigned to a single normal mode. A general approach towards composing the sought after ET dimension from the vibrational modes is to quantify how well aligned each mode is with the Marcus–Hush model. A linear combination of all vibrational modes should therefore result in a unique ET dimension, as proposed by Rudolph Marcus and refined in the Marcus–Hush model.^1^ We call this coordinate obtained from *ab initio* calculations the Marcus dimension in the following.

The correct combination of vibrational modes is obtained by a multi-component fit of all vibrational modes to the electron position for a Class III system, or to the excitation energy for a Class II system, see eqn (5a) and (5b), respectively. This procedure will account for all vibrational degrees of freedom. The independent variables in the multi-component fit are the displacements in the direction of the vibrational modes (*q*_*j*_) and the dependent variable is either the electron position (e_pos._^−^) or the excitation energy (Δ*E*^*D*_0_−*D*_1_^):

5a *b*_0_ + *b*_1_ × *q*_1,*i*_ + … + *b*_3*N*−6_ × *q*_3*N*−6,*i*_ = e_pos.,*i*_^−^

5b *b*_0_ + *b*_1_ × *q*_1,*i*_ + … + *b*_3*N*−6_ × *q*_3*N*−6,*i*_ = Δ*E*^*D*^_*i*_^_0_−*D*_1_^

The coefficients *b*_*j*_ are used to construct the Marcus dimension as a linear combination of vibrational modes, which are vectors of Cartesian displacements of individual atoms. The resulting Marcus dimension will therefore also take the form of a vector of Cartesian displacements.

**Fig. 5:**
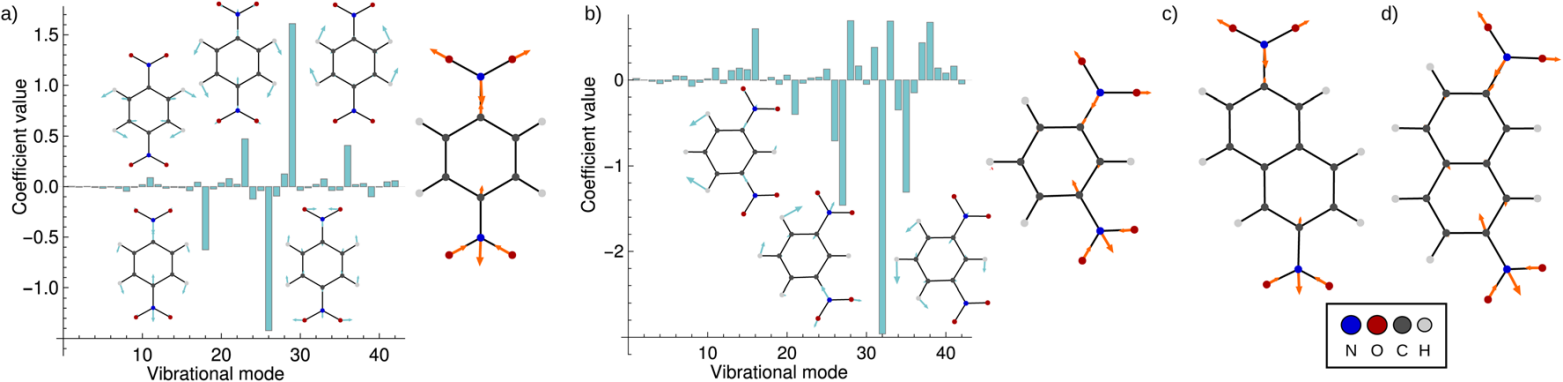
Coefficients obtained from the multi-component fit, including sketches of the normal modes with the largest coefficients. The resulting Marcus dimensions as linear combinations of the normal modes with expansion coefficients obtained from the multi-component fit are shown as sketches. (a) Results for *p*-DNB˙^−^, where the fit used the electron position according to eqn (5a). (b) Results for *m*-DNB˙^−^, where the fit used the excitation energy according to eqn (5b). (c) Obtained Marcus dimension for 2,6-DNN˙^−^ (fit using the electron position). (d) Obtained Marcus dimension for 2,7-DNN˙^−^ (fit using the excitation energy).

Fig. 5 shows the result of the fit for *p*-DNB˙^−^ (panel a) and *m*-DNB˙^−^ (panel b) as examples. By comparison of Fig. 5a with 4 we can again identify mode 29 as the most prominent motion for the Marcus dimension in *p*-DNB˙^−^. We emphasize that there are other modes with substantial contributions to the ET coordinate that are not expected among the chemically intuitive motions for the ET process. The role of these modes is thus to cancel out the motions of atoms that are not important for the Marcus dimension. The remaining modes have small, but non-zero, contributions which would be overlooked if the Marcus dimension was determined only from the correlation plots in Fig. 4.

The fact that many modes contribute in non-negligible amounts shows that the Marcus dimension should not be thought of as a single anti-symmetric normal mode. Therefore, it is not possible to fully equate the absorption in a specific infra-red or Raman region with the ET coordinate: some important contributors to the Marcus dimension may be infra-red or Raman silent, or they may appear outside of the region of interest. In addition, there may be irrelevant absorption bands in the energetic region of interest.

The obtained Marcus dimensions for the remaining dinitroradical anions are plotted in panels (c) and (d) of Fig. 5. The Marcus dimension is a motion localized on the nitro groups and the carbon atoms they are attached to. The most prominent parts of the motions are C–N bond stretches and N–O bond stretches. All motions are anti-symmetric which agrees with the ET coordinate described by the Marcus model,^51–53^ and very similar amongst each other, which is expected for structurally and electronically similar compounds. We emphasize here that our procedure elucidates the participation of each atom in the reaction coordinate without making any prior assumptions, other than assuming the validity of the Marcus–Hush model.

Upon repeating the whole procedure in vacuum, where all studied systems belong to Class III, qualitatively the same motions are obtained (see Fig. S3†). It is important to note that even though *m*-DNB˙^−^ and 2,7-DNN˙^−^ switch from a localized to a delocalized system, the Marcus dimension obtained by our approach is identical. The results support our ansatz of using the electron position or the excitation energy as dependent variables in the multi-component fit, which clearly gives equivalent results. Moreover, these findings provide additional confirmation that the motion identified with our procedure is the *intrinsic electron transfer coordinate* in the respective molecule.

### 5.3 Scan along the Marcus dimension

As further evidence that the dimensions found above correspond to those originally proposed in the Marcus model and that the ET results from nuclear motion along this dimension, an unrelaxed scan was performed along the vibrational coordinate shown in Fig. 5. In practice, the Marcus dimension is obtained as a vector of Cartesian displacements from the equilibrium configuration. This vector can be normalized and progress along the Marcus dimension can be measured in terms of the displacement of the nuclei from their equilibrium position.

**Fig. 6:**
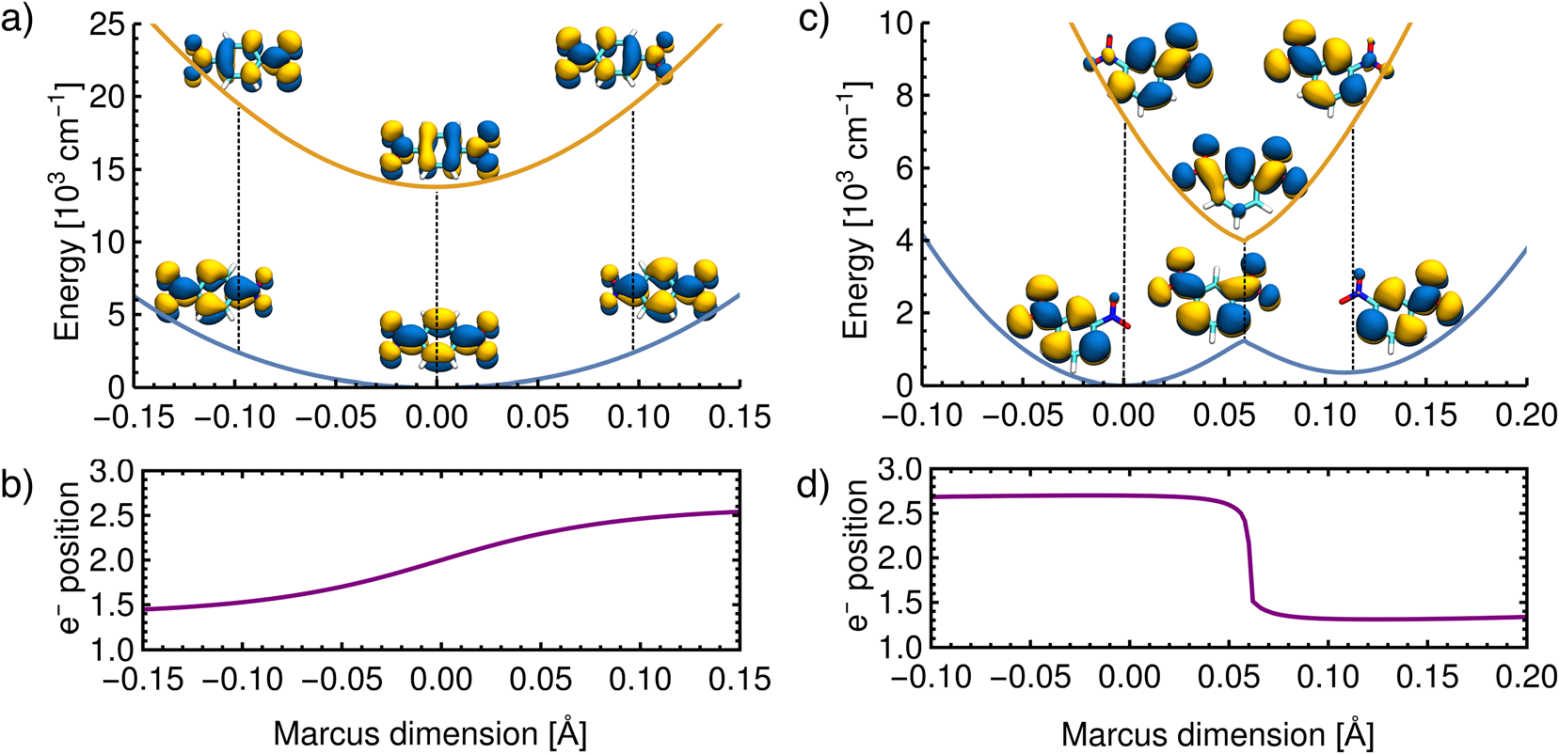
Reconstructed potential energy curves from geometries displaced in the direction of the Marcus dimension using the electronic energies. (a) Results for *p*-DNB˙^−^ in ACN. The scan was performed with a step size of 0.005 Å. (b) The electron position for *p*-DNB˙^−^. (c) Results for *m*-DNB˙^−^ in ACN. The scan was performed with a step size of 0.002 Å. (d) The electron position for *m*-DNB˙^−^. The change of the electron position in the ground and excited states is illustrated in the upper panels using natural transition orbitals and quantified in the lower panels according to eqn (3).

First, we show the results for *p*-DNB˙^−^, a Class III system. The potential energy surfaces of the ground and the first excited states are plotted in Fig. 6a, where the localization or delocalization of the unpaired electron is illustrated by natural transition orbitals. The scan results in two nested parabolic-like surfaces with different curvatures. The adiabatic minimum is characterized by an electron delocalized over the entire molecule. Any motion along the Marcus dimension will lead to a degree of localization of the electron on one of the nitro groups. This localization is reversed in the excited state. We therefore conclude that the system behaves according to Marcus theory of ET and that the identified ET coordinate is correct.

Moving on to a Class II system, *m*-DNB˙^−^ in ACN (see Fig. 6c), the ground state energy profile is a double-well potential and the excited state is a harmonic-like potential with the minimum centered just above the top of the ground state barrier. The qualitative agreement of the *ab initio* energy profiles with the Marcus model is a necessary, but not sufficient, condition to demonstrate that this dimension is the ET coordinate. The motion along the Marcus dimension should result in a change of the diabatic state. In other words, by overcoming the ground state barrier the unpaired electron has to be transferred to the other center. At the equilibrium configuration (position 0 Å in Fig. 6d), the unpaired electron is localized on the left-hand side nitro group in the ground state and on the right-hand side one in the excited state. At the top of the barrier, the unpaired electron is delocalized over the entire molecule. Once the barrier is overcome, the electron is localized on the right-hand side nitro group in the ground state and on the left-hand side one in excited state. This behaviour agrees exactly with the Marcus–Hush model, and hence the Marcus dimension we obtained from the multi-component fit is the dimension that facilitates electron transfer.

There are, however, two aspects in the Class II case that display some deviations from the Marcus model: there is a cusp at the top of the barrier, and the double-well potential is not symmetrical. These two aspects are discussed below.

The scan in Fig. 6c shows that the ground state potential does not change smoothly at the top of the barrier; instead, it exhibits a cusp. The same is seen in the excited state where the energy minimum takes the shape of a cusp. This appears to be a direct result of our approach, considering that the size of the scan steps is 0.002 Å and it is therefore unlikely that the curvature was missed due to a too widely spaced grid. This observation does not agree with the Marcus model, where the potential curves are smooth everywhere. The top of the barrier is in the region of an avoided crossing so that a multideterminant method might be necessary. In the ESI,† we compare the scans of the potential energy curves obtained from DFT with wavefunction methods (CASSCF, CASSCF/NEVPT2), showing that the cusp might be an artifact of using a single reference method. However, a proper description of the potential curves requires a strongly correlated method due to the expected importance of dynamic correlation. This is relatively easily captured with an appropriately chosen density functional, but when using wavefunction methods it appears that very large active spaces in combination with a perturbative treatment will be needed.

**Fig. 7:**
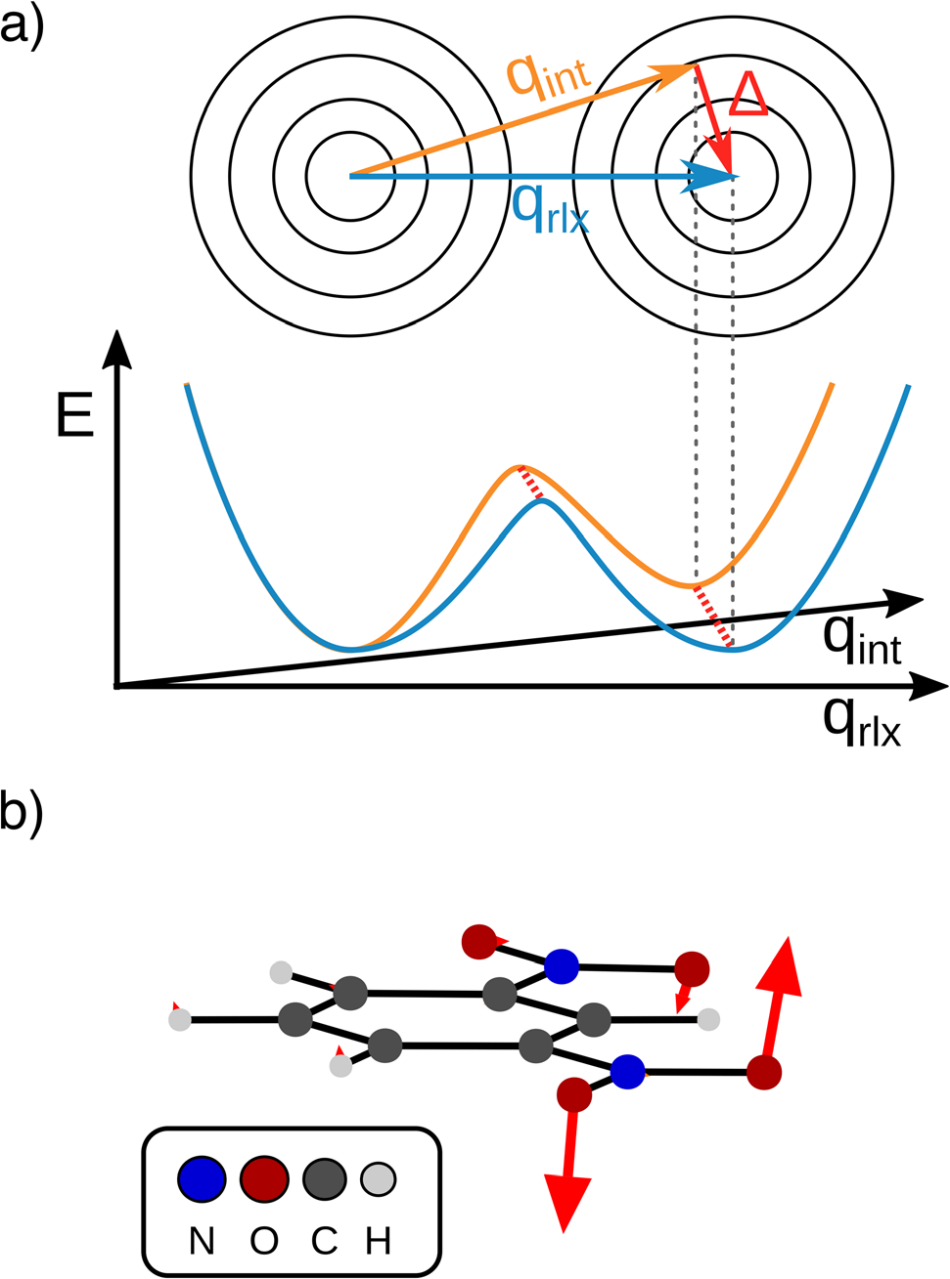
(a) Schematic representation of the separable intrinsic ET coordinate (Marcus dimension, *q*_int_, orange) and orthogonal structural relaxation (Δ, red arrow) *vs.* the relaxed ET coordinate (*q*_rlx_, blue) that may be obtained from a LICC approach. (b) Structural relaxation obtained by LICC between the second minimum at the Marcus dimension and fully optimized geometry.

We now move on to discuss the energetic asymmetry in the double-well potential shown in Fig. 7c. Since the *m*-DNB˙^−^ is a symmetrical system, both adiabatic minima in the double-well potential must have the same energy. The initial minimum (position 0 Å in Fig. 6c) was found by geometry optimization and is therefore described properly. Displacing the molecular geometry along the Marcus dimension results in the second minimum which is 350 cm^−1^ higher in energy. Of course the obtained potential could be symmetrized easily, but we do not expect to gain any additional insight from a symmetrized potential. In fact, we interpret the offset between the minima as the stabilisation that would be achieved by nitro group twisting, which implies that the electron transfer itself does not require twisting of the nitro group (see Fig. 5).

In other words, the Marcus dimension contains only the motions that directly promote the ET and neglects the motions that lead to the most stable conformer: our approach separates the intrinsic electron transfer coordinate from structural relaxation and environmental influences. This idea is illustrated in Fig. 7a, showing the Marcus dimension *q*_int_ as an orange arrow and an orthogonal relaxation to reach the second adiabatic minimum as a red arrow. The blue arrow *q*_rlx_ represents the direct, relaxed connection of the adiabatic minima, which would be obtained from a linear interpolation of Cartesian coordinates (LICC) and includes motions that do not contribute to electron transfer (here: twisting of the nitro groups). The separability of the intrinsic ET dimension from orthogonal structural relaxations is demonstrated in Fig. 7b. A linear interpolation from the endpoint of the intrinsic electron transfer coordinate to the optimized minimum, *i.e.* the motion associated with the red arrow, is found to be exclusively nitro group twisting and thus confirms the above interpretation. This finding is readily rationalised: in the initial minimum, the π system of the donor nitro group with the unpaired electron is conjugated with the π system of the benzene ring; only once the unpaired electron has been transferred to the acceptor nitro group can the system gain energy by rotation about the C–N bond to again enable stabilisation *via* π system conjugation. While for the cases studied here, there clearly are two separable motions, one can imagine cases where the intrinsic coordinate directly connects the two minima and no significant structural relaxation is seen, *e.g.* when the electron is placed in a non-bonding orbital at the acceptor site. The offset of the two minima discussed in Fig. 6c is then explained by choosing an adiabatic minimum which is a relaxed structure as a reference.

A more detailed comparison of the motions obtained by LICC *vs.* our approach is shown in the ESI.† We note here that the barrier of the Marcus dimension is *ca.* 200 cm^−1^ higher than that obtained from LICC. This relatively small difference supports the above reasoning that our approach does not reveal the minimum energy pathway. This is not a surprising result since the only constraint enforced in the fit is a correspondence to the Marcus–Hush model.

Our findings suggest that the minima along *q*_int_ are actually spatially very close to each other in the many-dimensional space of atomic nuclei: they have to move by only 0.12 Å to reach the other adiabatic minimum. In contrast, the barrier obtained along the LICC path is wider (0.2 Å), again in line with separability of the intrinsic ET dimension from unrelated structural relaxation as shown schematically in Fig. 7a. Considering that the barrier is rather thin and the height agrees with literature expectations,^55,56^ heavy-atom tunneling might be an important process for the ET. This has been discussed in early work,^57–59^ and modern measurements found that heavy-atom tunneling in organic compounds to be important for many reactions.^60–62^ It may therefore be worthwhile to reconsider the importance of heavy-atom tunneling for intramolecular electron transfer. For the two Class II systems studied here, we estimated the transmission coefficients through the barrier as 0.34 for *m*-DNB˙^−^ and 0.13 for 2,7-DNN˙^−^, respectively (see ESI† for details). These perhaps unexpectedly high values suggest that ET might be observable even at very low temperatures. In contrast, the transmission coefficients through the barrier obtained by LICC are significantly lower (*m*-DNB˙^−^: 0.04). Having a tool at hand that facilitates the evaluation of whether heavy-atom tunneling is relevant for intramolecular ET is an additional demonstration of the utility of being able to specify the ET coordinate and to quantify the electronic structure evolution along it.

### 5.4 Parameterization of the Marcus model

With the procedure described above, the Marcus model can now be recovered in the potential obtained from an *ab initio* calculation. To this end, an analytical potential is parameterized to the Marcus–Hush theory so that it matches as closely as possible to the *ab initio* potential. Before discussing the results, we note that in view of the idea in Fig. 7a, the experimental findings may be dependent on the experimental conditions, *i.e.* does the experiment allow the system to thermally relax, measuring the ET along the blue and red arrows in Fig. 7a, or is the measurement sufficiently rapid to separate intrinsic ET and vibronic relaxation?^63^

**Fig. 8:**
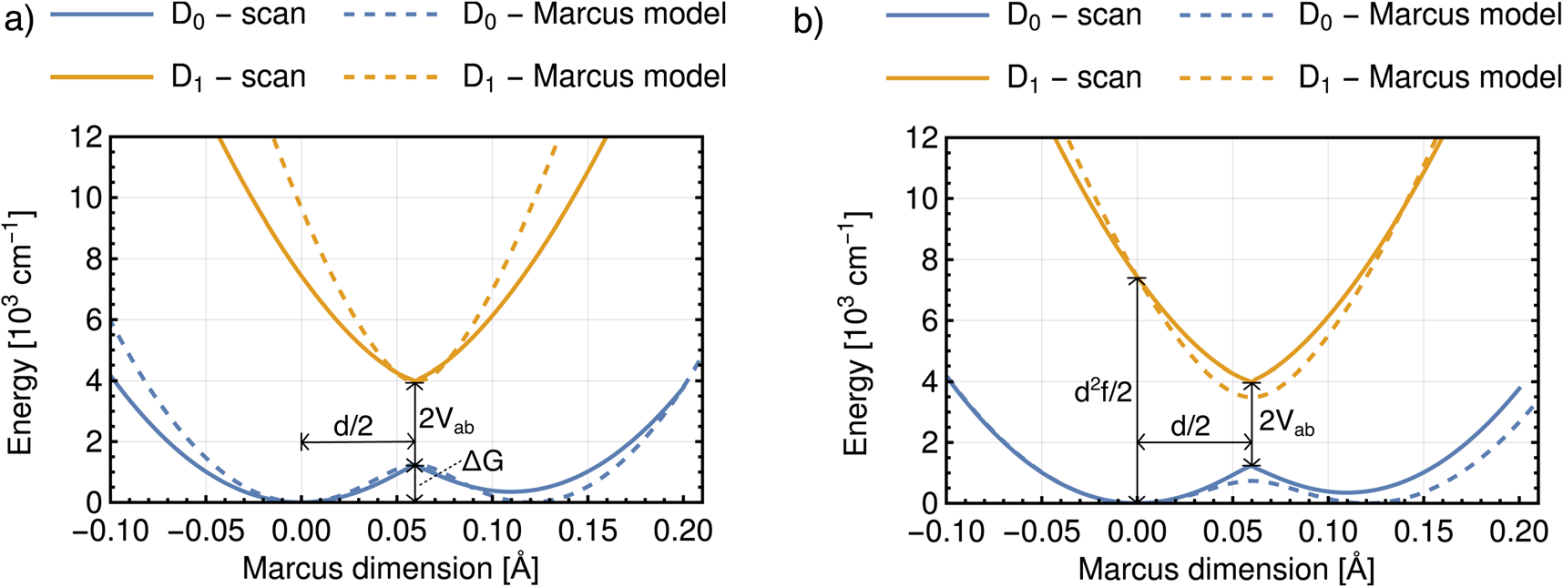
Potential energy curves obtained from the scan and from the parameterized Marcus model with two different parameterization procedures. As input parameters were used: (a) the excitation energy at the top of the barrier, the height of the barrier and the distance from the left adiabatic minimum to the top of the barrier; (b) the excitation energy at the top of the barrier, the excitation energy at the adiabatic minimum and the distance from the left adiabatic minimum to the top of the barrier. Results for *m*-DNB˙^−^ in ACN.

There are four quantities that ultimately characterize the *ab initio* potential energy surfaces: (i) the excitation energy at the adiabatic minimum, (ii) the height of the barrier, (iii) the excitation energy at the top of the barrier, and (iv) the distance between the adiabatic minimum and the top of the barrier. These are depicted in both panels of Fig. 8. The Marcus model has three parameters: the potential coupling *V*_ab_, the separation of the minima of the diabatic states *d*, and their force constants *f*. Only the potential coupling 2*V*_ab_ can be read immediately from the scan; it is the excitation energy at the top of the barrier. With the knowledge of the potential coupling we can relate other quantities obtained from the scan to the Marcus model. A more detailed discussion on how to choose the input values for the parameterization and description of the entire procedure is given in the ESI.†

The resulting parameters of the Marcus model will depend on the choice of parameterization procedure (sketched in Fig. 8). If the parameterization uses the potential coupling, the height of the barrier, and separation of the adiabatic minimum from the top of the barrier (parameterization A, Fig. 8a), the obtained potential energy curves will agree better with the ground (D_0_) than with the excited state (D_1_), see Fig. 8a. This is due to the fact that the reorganization energy from the *ab initio* scan does not enter the parameterization. In contrast, if the parameterization uses the reorganization energy, the separation of the adiabatic minimum and the top of the barrier, and the potential coupling (parameterization B, Fig. 8b), the shape of the potential curves for both ground and excited state will be better represented. However, the height of the barrier will not agree with the *ab initio* potential.

The results illustrate that the Marcus model faithfully captures the general shape of the potential curves but does not result in quantitative agreement with the *ab initio* potential. This is reflected in the parameters obtained for the barrier height Δ*G* and the reorganization energy *λ*, see Table 1. A disagreement might be expected given the offset between intrinsic and relaxed ET coordinates, and that the Marcus model assumes harmonic diabatic states which might not be the case in a real system for which the anharmonicities of the diabatic states enter the *ab initio* potential. In addition, the electronic structures at the top of the barrier are not described perfectly, but since the error introduced by the electronic structure method cannot be avoided or removed, the predicted shapes of the potential energy curves cannot be expected to agree exactly with the shapes in the Marcus model. We note that parameterizing the Marcus model for a specific system is not a straightforward procedure; in most cases it can be expected that a compromise between describing well either the barrier height or the reorganisation energy will need to be found to best represent the system.

**Table 1:** Results from parameterization of the Marcus model. Rows labelled A and B correspond to the parameterization procedure illustrated in Fig. 8a and b, respectively

	Class II system	Δ*G* [cm^−1^]	*λ* [cm^−1^]	2*V*_ab_ [cm^−1^]	*d* [Å]
A	*m*-DNB˙^−^	1233	9652	2752	0.125
	2,7-DNN˙^−^	1644	10 386	2111	0.135
B	*m*-DNB˙^−^	737	7434	2752	0.129
	2,7-DNN˙^−^	970	7510	2111	0.138
*Ab initio* scan	*m*-DNB˙^−^	1233	7434	2752	0.120
	2,7-DNN˙^−^	1644	7510	2111	0.132

Perhaps the most surprising result is the separation of the diabatic states *d*, which is unexpectedly low in all cases. Prior experimental studies of dinitroradical anions^40,64^ made use of the generalized Mulliken–Hush formula^12^ to estimate the donor–acceptor separation from the transition dipole moments, evaluated either experimentally^64^ or by using semi-empirical calculations.^40^ The values ranged from 2 to 6 Å and it was noted that these were significantly shorter than the distance between the redox centers.

At first glance, such a discussion of distances raises the question whether we need to resolve the fact that these distances are much longer than the distances on the order of tenths of an Ångström we find. We note that the distances measured by dipole moment are fundamentally different from the ones we find by *ab initio* scans. The former are attempts at measuring the ‘real-space’ distance of electron transfer, while our approach identifies the distance in a multidimensional space expressed as the Marcus dimension. Nevertheless, one might be tempted to ascribe the results obtained from transition dipole moments to the distance along the Marcus coordinate. However, a simple thought experiment can show this is not reasonable. If we choose even the shortest distance (*d* = 2 Å) and use reasonable values for the reorganization energy 
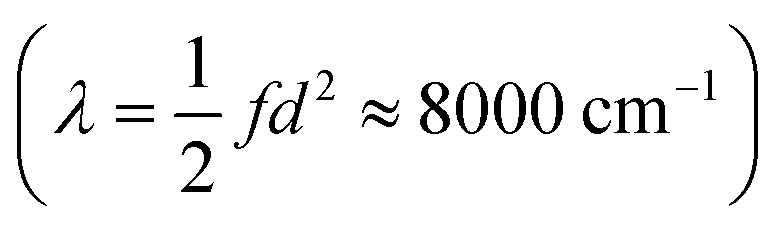
 and coupling (2*V*_ab_ ≈ 2500 cm^−1^),^23,55,65^ the remaining parameter is the spring constant *f* which becomes 11 kcal mol^−1^ Å^−2^. This is a unreasonably small spring constant that would result in an extremely low frequency along the ET coordinate. For instance, if we assume only a single N atom is moving, we can ascribe a mass of 14 amu, and the spring constant yields a frequency of 100 cm^−1^. On the other hand, our separations are associated with frequencies on the order of 1000 cm^−1^, which is in better agreement with expectations for the nuclear motions associated with ET.

### 5.5 ET rate constant

In systems that conform to the Marcus model, the ET rate can in principle be assessed with the Marcus equation (eqn (1)).^3,4^ The expression is based on Fermi's golden rule and is thus valid only for small values of the potential coupling *V*_ab_. Another assumption employed in the Marcus equation is that all vibrational motions involved in the ET are small in frequency (ℏ*ω* ≪ 2*k*_B_*T*); this is the so-called high temperature limit.^3^ The Marcus equation is widely used to predict the ET rate in proteins,^66^ the rate of the photoinduced ET,^67^ or the rate of the inter-system crossing.^68^

For large potential couplings *V*_ab_, the ET can be assumed to happen only adiabatically, *i.e.*, once the nuclei reach the top of the barrier the probability of the electron tunneling is close to one.^4^ In this case, the pre-exponential factor will reduce to a nuclear frequency along the ET coordinate *ν*_*q*_ and the ET rate can be calculated according to eqn (2). The nuclear frequency *ν*_*q*_ is a well defined concept only in the harmonic potential. In the double-well potential this value has to be estimated, and we propose to use the harmonic approximation at the adiabatic minimum to obtain the force constant and subsequently the harmonic frequency (see Sec. S7† for more details). Regardless of how *ν*_*q*_ is estimated, the value is approximately 10^13^ s^−1^.

A comparison of ET rates according to the Marcus equation with the adiabatic rates is shown in Table 2. The adiabatic, *i.e.* classical, rate was computed using the barrier height from the scanned potential and the harmonic frequency at the adiabatic minimum. The parameters for the Marcus equation were taken from parameterization procedure A.

**Table 2:** ET rate constants in 10^10^ s^−1^ for Class II systems in ACN at 300 K. The classical rate is computed using eqn (2). The Marcus equation refers to eqn (1) with input values taken from parameterization scheme A

	*m*-DNB˙^−^	2,7-DNN˙^−^
Classical	8.16	1.22
Marcus eqn	125.7	9.52
Experiment	4.63^a^^,^^b^	0.31^c^

a Value from ref. 56.

b Telo *et al.*^55^ reported value 1.54 10^10^ s^−1^.

c Value from ref. 40.

The ET rates listed in Table 2 favor the description with the more simplistic eqn (2) over that with the Marcus equation (eqn (1)). We can identify two reasons for this finding: (I) both systems under study have large potential coupling values *V*_ab_, which are outside the scope of the Marcus equation. (II) The nuclear frequency in the Marcus dimension is larger than 2*k*_B_*T*. Note that the Marcus dimension is confined mainly to the stretching of C–N and N–O bonds which have frequencies of *ca.* 1000 cm^−1^.

For systems with large potential coupling *V*_ab_, the ET mechanism is adiabatic and the ET rate should be assessed classically (*i.e.*, with eqn (2)). Since the biggest error is introduced by the electronic structure method, choosing a different method of estimating the nuclear frequency *ν*_*q*_ would improve the value of the ET rate only through error cancellation. Classical treatment of ET is only possible for high temperatures; heavy-atom tunneling which might drive ET at low temperatures should in principle be possible due to the very narrow barrier. Another case where the classical theory will fail is a photoinduced ET which is, however, beyond the scope of this paper.

## 6 Conclusions

We propose a method for identifying the nuclear coordinate that promotes electron transfer in mixed valence systems by exploiting properties postulated by Marcus–Hush theory in the analysis of a thermally representative ensemble of *ab initio* calculations. As the characteristic electron transfer property, the electron position is chosen for Class III systems with complete delocalisation of the unpaired electron, and the excitation energy is chosen for Class II systems where a small barrier separates two adiabatic minima with localisation of the unpaired electron on one of the redox centers. The electron transfer coordinate, also termed Marcus dimension, is obtained as a linear combination of vibrational modes. We find that the Marcus dimension is not simply equivalent to the absorption in a certain IR or Raman region; the *ab initio* analysis shows that active and silent modes across all energies contribute.

The method was demonstrated on a set of organic radical compounds with two nitro groups as the redox centers. The Marcus dimension was found to be a chemically intuitive antisymmetric motion mostly restricted to the redox centres (nitro groups) and the atoms of the bridging unit (aromatic core) that they are attached to. The motion is qualitatively very similar for structurally similar compounds, and is retained even after an environmentally induced change from Class II (*m*-DNB˙^−^ and 2,7-DNN˙^−^ in ACN) to Class III (both in vacuum). We have thus shown that our approach identifies the nuclear coordinate that is the electron transfer pathway in Class II compounds and leads to a higher degree of charge localization in Class III compounds. Scans along the Marcus dimension clearly demonstrate that the expected potential shapes are obtained, and furthermore provide the basis for recovering the Marcus model from *ab initio* calculations. To the best of our knowledge, this is the first approach for identifying the electron transfer coordinate that is applicable across all Robin–Day Classes.

The Marcus dimension we identify for Class II compounds appears to be an intrinsic property of the molecule, which implies separability of the transition from one adiabatic minimum to the other into the electron transfer process and ET-innocent structural relaxations or environmental contributions. This is demonstrated explicitly for the examples studied here and by comparison with the previously employed linear interpolation of Cartesian coordinates approach. The usefulness of the presented approach lies in extracting the exact nuclear motion that is responsible for promoting ET, which does not have to coincide with the lowest energy pathway. It may be possible to devise an experimental test for our suggestion of separable electron transfer and structural relaxation events, *e.g.* using frozen solution or solid state experiments that prevent structural relaxation and/or ultrafast spectroscopies that probe electronic structures prior to structural relaxation.^63^

The *ab initio* scans along the Marcus dimension show a small separation of the minima. The parameterization of the Marcus model provides acceptable agreement with experiment, and we note that other approaches may produce better results^22,25^ especially for weakly coupled systems. The parameterization results in similar separations of the diabatic states of *ca.* 0.12–0.13 Å, *i.e.*, much thinner barriers for electron transfer than had been previously suggested. This small distance in the multi-dimensional space of atomic nuclei suggests that heavy-atom tunneling may be a relevant contributor to intramolecular electron transfer. We estimated transmission coefficients of 0.34 for *m*-DNB˙^−^ and 0.13 for 2,7-DNN˙^−^. Low temperature experiments may confirm this finding in future, which would be one way of obtaining experimental support for the concept of the Marcus dimension introduced here.

With a method for identifying the Marcus dimension of electron transfer in any mixed valence system for which TD-DFT calculations on a representative ensemble can be obtained, a more rigorous and quantitative discussion of electron transfer is now possible, likely also extending to proton-coupled electron transfer. It provides an opportunity to evaluate ET mechanisms involving molecular orbitals or electronic states proposed in the literature, *e.g.* incoherent hopping *vs.* coherent superexchange mechanisms, or two-state *vs.* multi-state models.^6,54^ Our approach is the first to provide the spring constant of the nuclear motion and the separation of the adiabatic minima. Individual access to all parameters of the Marcus model holds promise for disentangling electronic structure behaviour arising from the electronic coupling, the curvature of the PES and the separation of the minima, as well as decoupling and quantifying the intramolecular and solvent contributions to the ET coordinate.

## Data availability

We provide extensive ESI† and have now made available the script used to fit the data and generate the Marcus dimension, https://git.rwth-aachen.de/ak-krewald/mdlcnm.

## Author contributions

Conceptualization: V. K., B. J. L. and A. S.; data generation: A. S.; analysis and interpretation: A. S., B. J. L. and V. K.; writing: V. K., B. J. L. and A. S.; supervision: V. K.

## Conflicts of interest

There are no conflicts to declare.

## Supplementary Material

SC-014-D3SC01402A-s001
