# Supplementary material for: The Marcus dimension: identifying the nuclear coordinate for electron transfer from ab initio calculations
Source: Chem Sci. 2023 Aug 8;14(34):9213–25. doi: 10.1039/d3sc01402a (PMC10466304; doi:10.1039/d3sc01402a)
Supplement: SC-014-D3SC01402A-s001 [file SC-014-D3SC01402A-s001.pdf]

Electronic Supplementary Material (ESI) for Chemical Science.
This journal is © The Royal Society of Chemistry 2023

The Marcus dimension: identifying the nuclear coordinate for electron transfer from *ab initio* calculations

Adam Šrut[a], Benjamin J. Lear[b*], Vera Krewald[a*]

Email: bul14@psu.edu; vera.krewald@tu-darmstadt.de

[a]TU Darmstadt, Department of Chemistry, Theoretical Chemistry, Alarich-Weiss-Straße 4, 64287 Darmstadt, Germany; [b]The Pennsylvania State University, Department of Chemistry, University Park, PA 16802, USA

Contents

1 IVCT spectra 2

2 ET driven by vibrational modes 4

3 Marcus dimensions 5

4 Conventional approach: linear interpolation of Cartesian coordinates 6

5 Multideterminant calculations 8

6 Scans along the Marcus dimension (acetonitrile) 11

7 Properties of the Marcus dimension 12

8 Parameterization of the Marcus model 13

9 Heavy atom tunneling 14

10 Composition of adiabatic states from Marcus–Hush theory 15

11 Cartesian coordinates of the adiabatic minima and Marcus dimensions in acetonitrile 16

1 IVCT spectra

To connect the electronic structures with experimental information, the IVCT bands were reconstructed from the nuclear ensemble method as shown in Fig. 1. The spectra were computed according to:[1]

$$\epsilon(E) = \frac{N_\mathrm{A} e^2}{4\ln(10) m_e c^2 \epsilon_0} \int_0^\infty \left[\sum_{j=1}^{N_\mathrm{geoms}} \sum_{i=1}^{N_\mathrm{states}} f_{ij} \cdot \delta(E' - E_{ij}) \right] \cdot g \left(- \left(\frac{E - E'}{0.6 \cdot 0.5\,\mathrm{eV}} \right)^2 \right) \mathrm{d}E', \quad (1)$$

where N_A is the Avogadro constant, e is the elementary charge, c is the speed of light, m_e is the mass of the electron, ϵ_0 is the permittivity of vacuum, δ is the Dirac delta function, g is the normalized Gaussian line shape with full width at half maximum 0.5 eV, f_{ij} and E_{ij} are oscillator strength and excitation energy for i-th electronic state and j-th geometry in the ensemble, respectively.

We compare the results for the electronic structures computed with the local hybrid density functional LH20t[2] with those obtained with the BLYP35[3] functional proposed previously that is based on a global hybrid functional (BHLYP[4] with 35 % exact exchange). The BLYP35 calculations were run with the ORCA[5] suite of programs employing the def2-TZVP[6] basis set for carbon, oxygen and nitrogen atoms and def2-SVP for hydrogens. Solvation with ACN was modelled implicitly using a polarizable continuum model. The resolution of identity approximation was employed to approximate Coulomb and exchange integrals.[7] Convergence criterion for the self-consistent field procedure was set to $10^{-8}\,E_\mathrm{h}$.

Both electronic structure methods show a reasonable performance in predicting the IVCT bands. We note in particular that the relative intensities for the Class II and Class III cases are reproduced well; no scaling factors are applied in the comparison of experiment and theory. The characteristic spikes in the experimental spectra of Class III compounds are not reproduced in the computational spectra. A possible explanation might be found in the approximated treatment of vibronic transitions in the nuclear ensemble method (the Duschinsky effect and the frequency shift between ground and excited state are neglected).

Of the two density functionals, LH20t predicts the band shapes better for all systems studied and yields slightly better intensities for the Class III cases (p-DNB$^{\bullet-}$ and 2,6-DNN$^{\bullet-}$). With BLYP35, the predicted bands are too wide, which might be due to a slightly too localized electronic structure or a not ideally described vibrational structure which in turn affects the sampling of geometries.

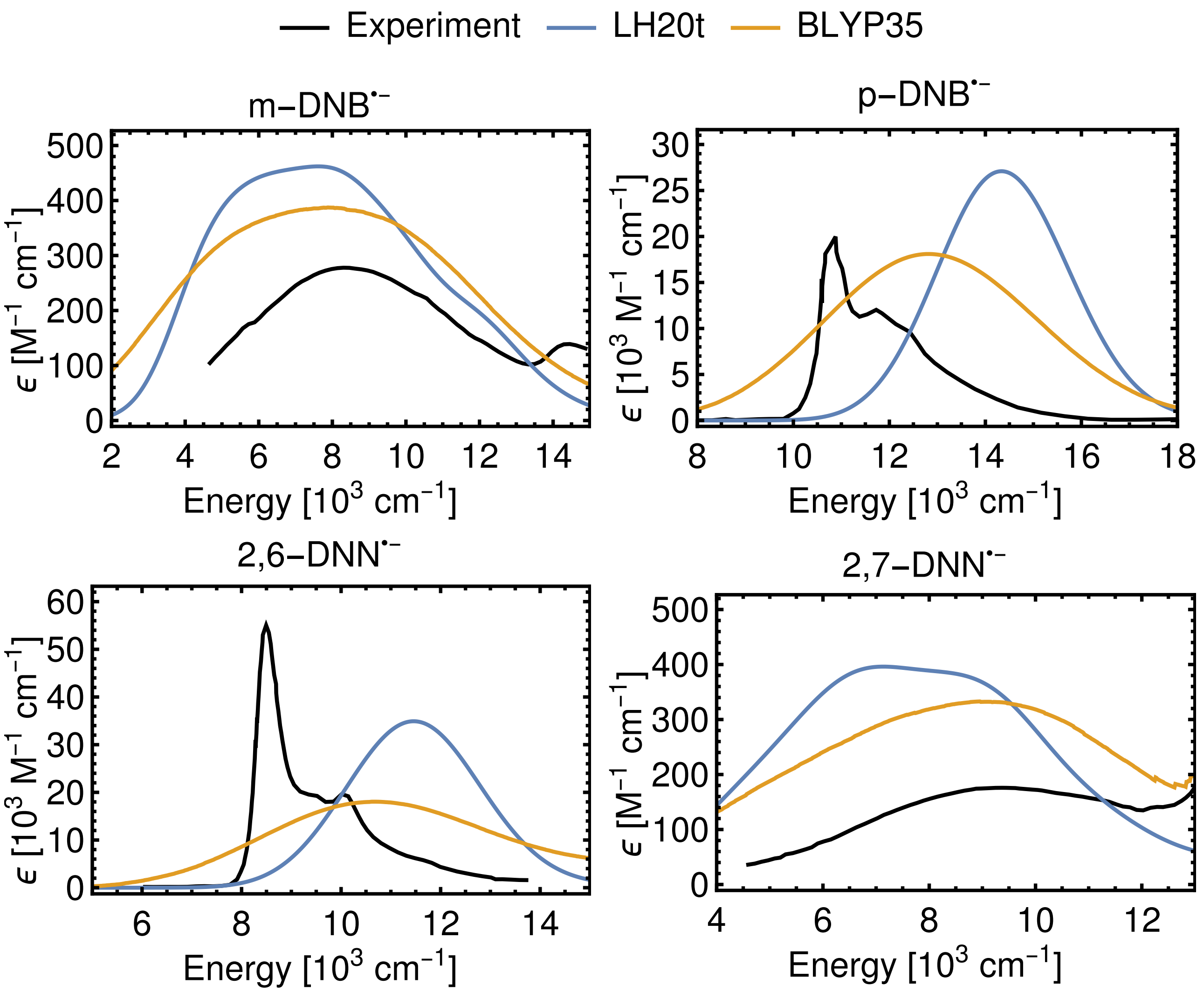

Figure 1: Computed IVCT bands of the Class II species *m*-DNB$^{\bullet-}$ and 2,7-DNN$^{\bullet-}$, and the Class III species *p*-DNB$^{\bullet-}$ and 2,6-DNN$^{\bullet-}$ using the nuclear ensemble method with the local hybrid density functional LH20t and the global hybrid density functional BLYP35 in ACN. Experimental spectra of *m*-DNB$^{\bullet-}$ and *p*-DNB$^{\bullet-}$ are taken from Ref. [8], and spectra of 2,6-DNN$^{\bullet-}$ and 2,7-DNN$^{\bullet-}$ from Ref. [9]. The spectrum of 2,6-DNN$^{\bullet-}$ was measured in dimethylformamide.

2 ET driven by vibrational modes

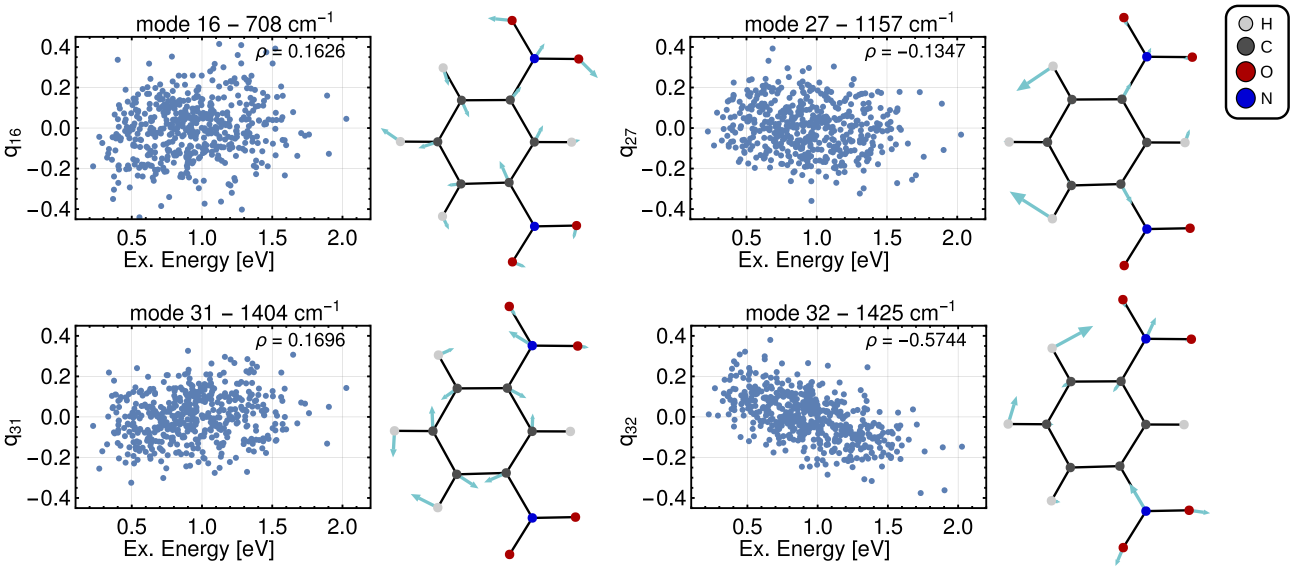

Figure 2: For 500 samples of the m-DNB$^{\bullet-}$ ensemble in ACN, the distortions along four selected normal coordinates are plotted against the excitation energy with a graphical representation of the corresponding vibrational mode. The plot labels show the index of the vibrational modes and their harmonic frequencies; the correlation coefficient ρ is given in the top-right corner of each plot. Other normal coordinates showed a lower correlation coefficient.

3 Marcus dimensions

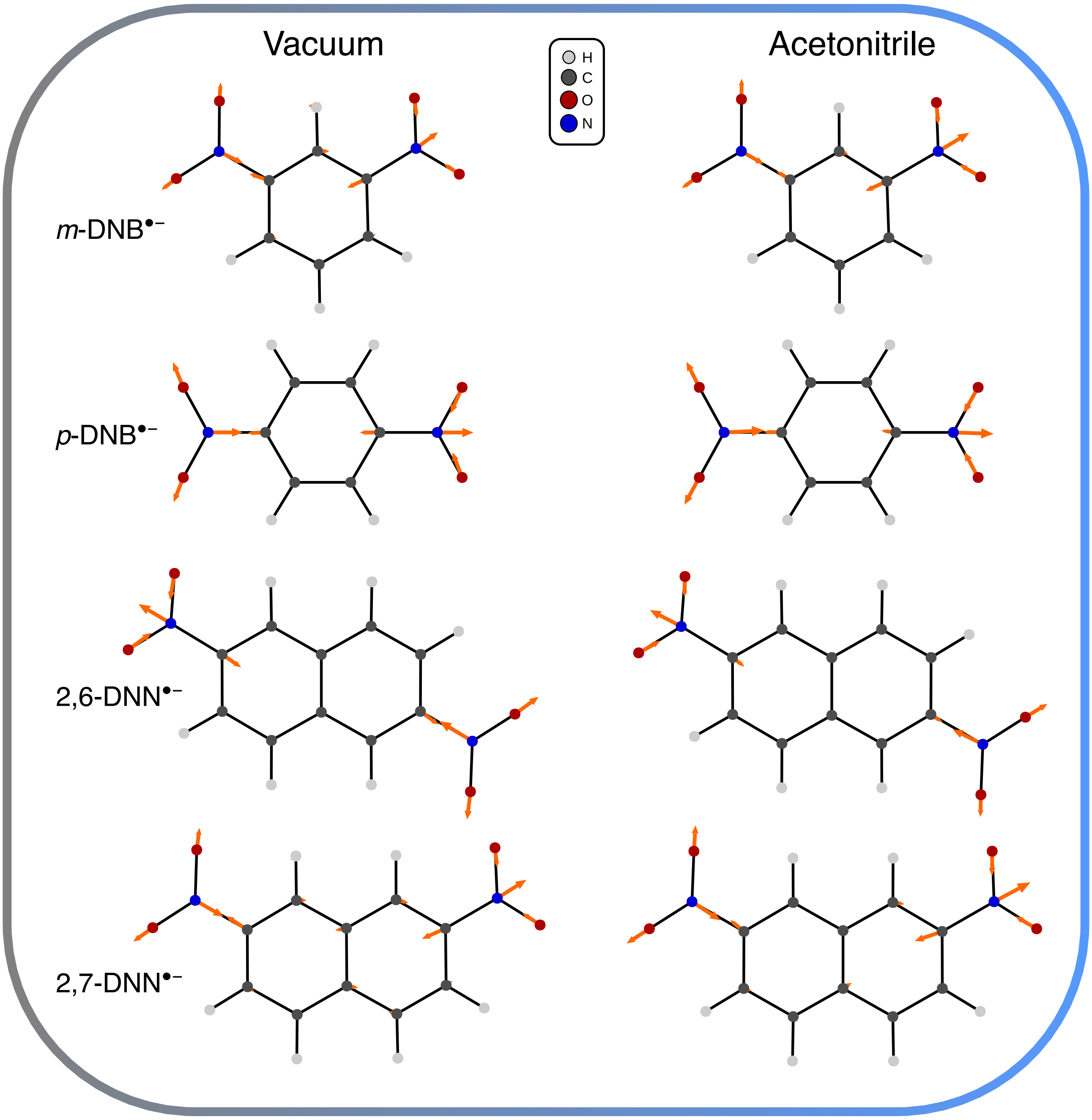

Figure 3: The electron transfer dimensions resulting from the multi-component linear fit as introduced in the main text for vacuum (left) and acetonitrile (right). In vacuum, all systems behave like a Class III system, and therefore the fit used the electron position. In acetonitrile, *m*-DNB$^{\bullet -}$ and 2,7-DNN$^{\bullet -}$ belong to Class II, and therefore the fit used the excitation energy to the lowest doublet state, whereas *p*-DNB$^{\bullet -}$ and 2,6-DNN$^{\bullet -}$ belong to Class III and the fit used the electron position.

4 Conventional approach: linear interpolation of Cartesian coordinates

In this Section we show the electron transfer dimension obtained from a linear interpolation of Cartesian coordinates (LICC) as has been used in the literature so far.[10] This method requires two well-defined structures in Cartesian coordinates, usually an adiabatic minimum and a totally symmetric structure representing the transition state. The reaction coordinate is obtained by linear interpolation between those structures.

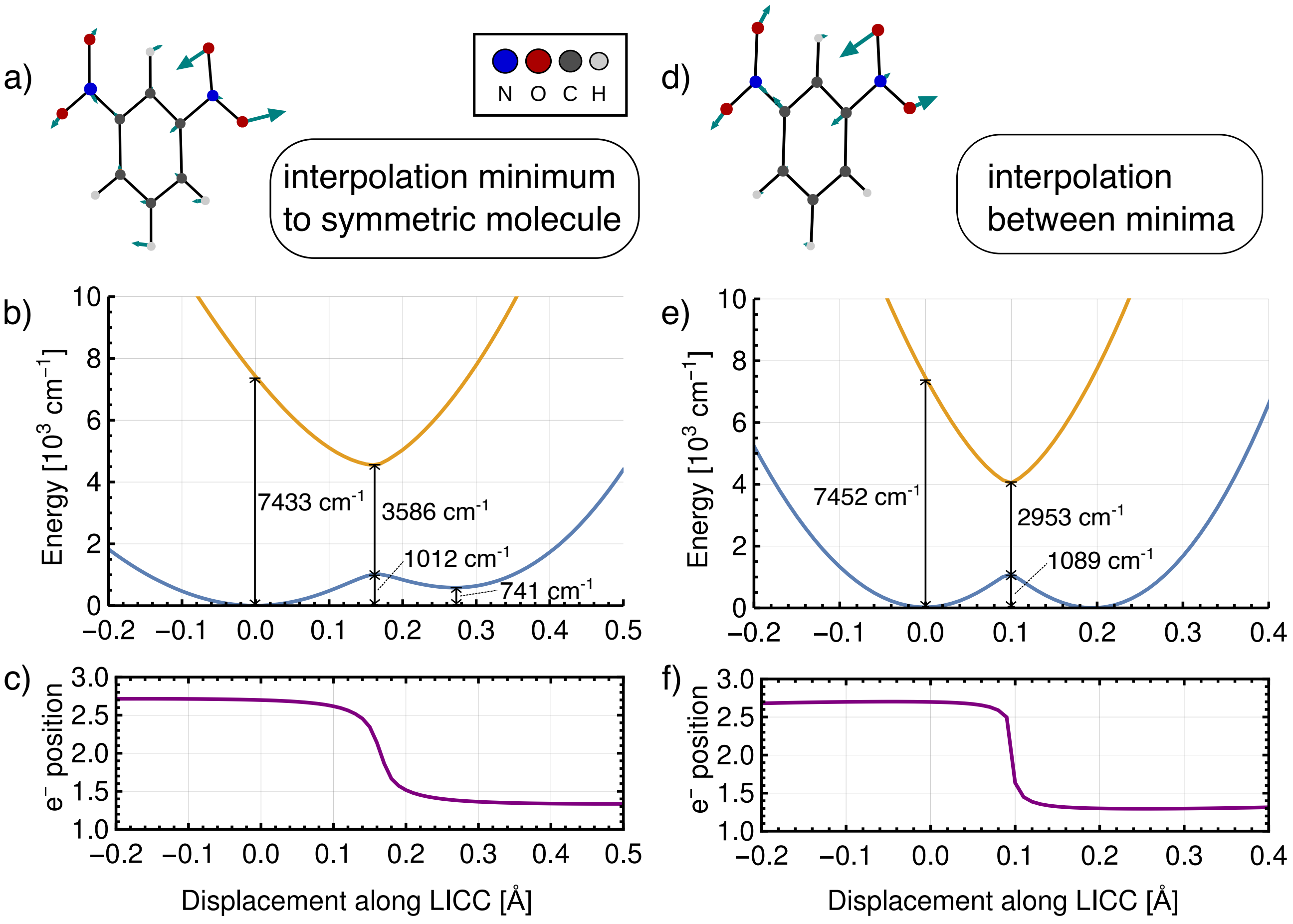

Figure 4: ET coordinates from the LICC approach and corresponding potential energy curves obtained from a scan along them. a) ET dimension obtained by LICC between the optimized geometry (adiabatic minimum) and the totally symmetrical one. b) Unrelaxed scan along the LICC dimension, with labels indicating the values of the reorganization energy, the electronic coupling, the height of the barrier, and the offset of the second minimum. c) Change of the electron position along the scanned coordinate. Panels d, e, f depict the same quantities for the ET coordinate obtained with LICC between the two adiabatic minima.

We note that this approach cannot be applied to Class III systems where only one well-defined structure exists. LICC cannot identify the ET dimension in Class III systems and could not be then used to decouple intramolecular and solvent motion. In addition, mapping the potential curves obtained by a LICC approach for a Class III system onto the diabatic (*i.e.* uncoupled) states of the underlying Marcus theory is thus precluded, and hence the progression of coupling strength, nuclear motion or force constants in a series of Class III systems or for borderline Class II/Class III cases could not be evaluated. Regardless of these conceptual limitations, we tested the LICC approach on m-DNB$^{\bullet-}$ in ACN. The LICC scans were performed between the adiabatic

minimum and the totally symmetric molecule (Fig. 4a), and between the two adiabatic minima (Fig. 4d), since these are the only well defined structures of a class II systems.

The ET dimensions obtained by LICC (Fig. 4a,d) are not anti-symmetric motions: a twisting motion of the oxygen atoms of one nitro group dominates the overall motion, and some hydrogen and carbon atoms of the aromatic ring have non-negligible contributions. In the adiabatic minimum, where the unpaired electron is localized on one side of the molecule (in this case, the nitro group on the left-hand side), the other nitro group is further away from the aromatic bridging unit and is slightly distorted, which will decrease the potential coupling with the bridging unit. A twisting motion is expected to be an important part of the ET only in cases of very large distortions.[11] For a nitro group perpendicular to the aromatic plane, the conjugation with the rest of the molecule breaks, and the potential coupling would thus tend to zero, making the ET adiabatically forbidden.

Unrelaxed scans along the two types of LICC coordinate (Fig. 4b,e) produce double-well potentials that change smoothly everywhere. For the LICC scan between the adiabatic minimum and the totally symmetric molecule, the minima do not have the same energy: the second minimum is destabilized by $741\,\mathrm{cm}^{-1}$, which is more than half of the ET barrier. The potential obtained by scanning between the two adiabatic minima is symmetric and the barrier height agrees well with the value obtained by simulating experimentally measured EPR spectra ($1000\,\mathrm{cm}^{-1}$).[8] The electron position changes upon overcoming the potential barrier in both cases (Fig 4c, f), with a sharper transition in the scan between the two adiabatic minima.

Clearly, the results of the LICC approach depend on the choice of the reference structures, and therefore it is unclear whether the ET coordinate obtained with a solvent model would lead to meaningful potentials when transferred into vacuum. Furthermore, a strong limitation of LICC is that it can only be applied to Class II systems, since for Class III systems no second geometric reference structure besides the adiabatic minimum exists. We will show in the following sections that by exploiting properties postulated in the Marcus model when interpreting the *ab initio* calculations, we arrive at a chemically intuitive dimension that drives electron transfer in Class II and leads to electron localization in Class III systems.

5 Multideterminant calculations

Seeking an explanation for the cusp observed in the scanned potential energy curve for m-DNB$^{\bullet-}$ using DFT, we repeated the scan with state-averaged complete active space self-consistent field (SA-CASSCF) calculations[12] and a subsequent treatment with the N-electron valence state perturbation theory (SA-CASSCF/NEVPT2).[13] The calculations were run with the ORCA[5] suite of programs employing the def2-TZVP[6] basis set and implicit solvation using a polarizable continuum model with acetonitrile as the solvent modeled. The resolution of identity approximation (RIJK) was employed to approximate Coulomb and exchange integrals.[7] Two electronic states were considered in the state-averaged calculations: the electronic ground and first excited state at the adiabatic minimum. The active space contains nine electrons in eight orbitals, *i.e.* (9, 8), which are selected from the conjugated π-system comprising the two nitro groups and the benzene ring. The remaining four π bonding orbitals were significantly lower in energy and not included in the active space. The orbital shapes change during the scan, see Fig. 7.

The scan along the Marcus dimension with CASSCF and CASSCF/NEVPT2 is shown in Fig. 5. The CASSCF approach provides unreasonably high excitation energies and the minima in the ground state potential curve are significantly shifted apart from each other. These are consequences of the lack of dynamical correlation, resulting in an overstabilized localized electronic structure. The CASSCF method results in a blue-shift of the IVCT band by more than 4000 cm^{-1} in comparison with the experiment. Accounting for dynamical correlation with the NEVPT2 approach (Fig. 5 right) leads to a substantial improvement of the potential energy curves. The double well potential in the ground state is correctly reproduced and the excitation energies match the position of experimentally measured IVCT band. The perturbation theory correction is, however, very large which may question the validity of the perturbative treatment in this case and calls for substantially enlarging the active space. This was tested on one structure by adding three low-lying orbitals from the conjugated π-system to the active space, *i.e.* (15, 11), which resulted in very similar energies and energy corrections to the electronic states. Since we are showing a proof of concept, enlarging the active space to achieve quantitative agreement is not the principal aim of this work. It can be reasonably expected that adding more orbitals besides the conjugated π-system considered here would enlarge the active space to the realms of a DMRG-SCF/NEVPT2 calculation.

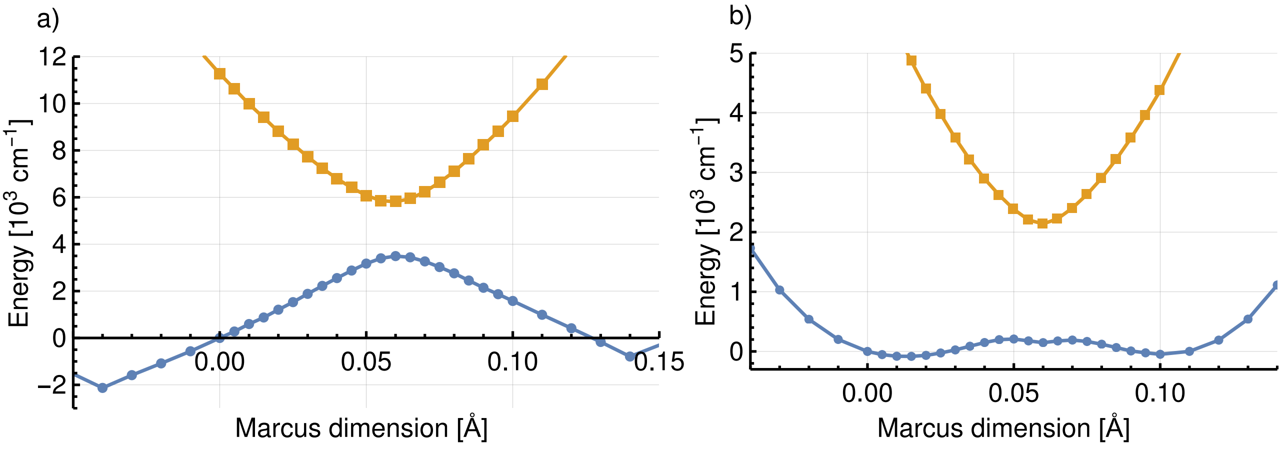

Figure 5: Scan along the Marcus dimension for m-DNB$^{\bullet-}$ in ACN with state-averaged CASSCF (a) and CASSCF/NEVPT2 (b) calculations using a (9, 8) active space.

The differences in the description of electronic structure with DFT and a multiconfigurational method are displayed in Fig. 6. The CASSCF/NEVPT2 method provides slightly lower excitation energies and the resulting IVCT band is thus expected to be red-shifted with respect to the experiment. The adiabatic minima of the CASSCF/NEVTP2 calculations are closer to each other than for the calculations with the LH20t density functional.

Most importantly, the scan with the wavefunction method results in a smooth shape of the potential barrier instead of the presumably unrealistic cusp. In fact, the barrier exhibits a feature exactly opposite to the cusp, a dip. This behaviour was already observed for other MV systems.[14] The dip can be explained as a stabilization of the wavefunction by delocalization, see Fig. 7. If the electronic structures adjacent to the dip are not as delocalized, they are expected to have higher energies.

The barrier height obtained from the CASSCF/NEVPT2 scan is, however, unreasonably small ($290\,\mathrm{cm}^{-1}$) which would result in an ET rate that is two orders of magnitude faster than the experimental value. We expect that a faithful description of the Marcus dimension can be achieved with a strongly correlated multireference method (*e.g.* MRCI or DMRG-SCF/NEVPT2 with a large active space). We suggest that DFT is prone to fail only in proximity of the very top of the barrier. While CASSCF/NEVPT2 delivers a smooth potential and a reasonable shape of the barrier, the absolute values obtained with the current active space are questionable as reflected in the unreasonably large corrections from the perturbative treatment.

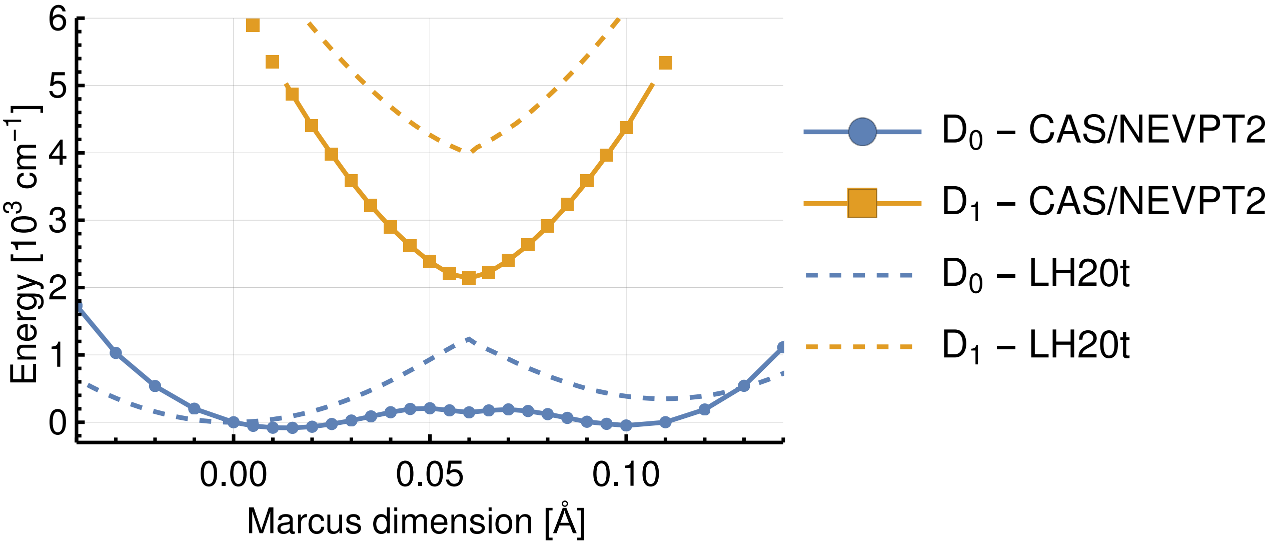

Figure 6: Comparison of state-averaged CASSCF/NEVPT2 and LH20t results for the scan along the Marcus dimension of *m*-DNB$^{\bullet-}$.

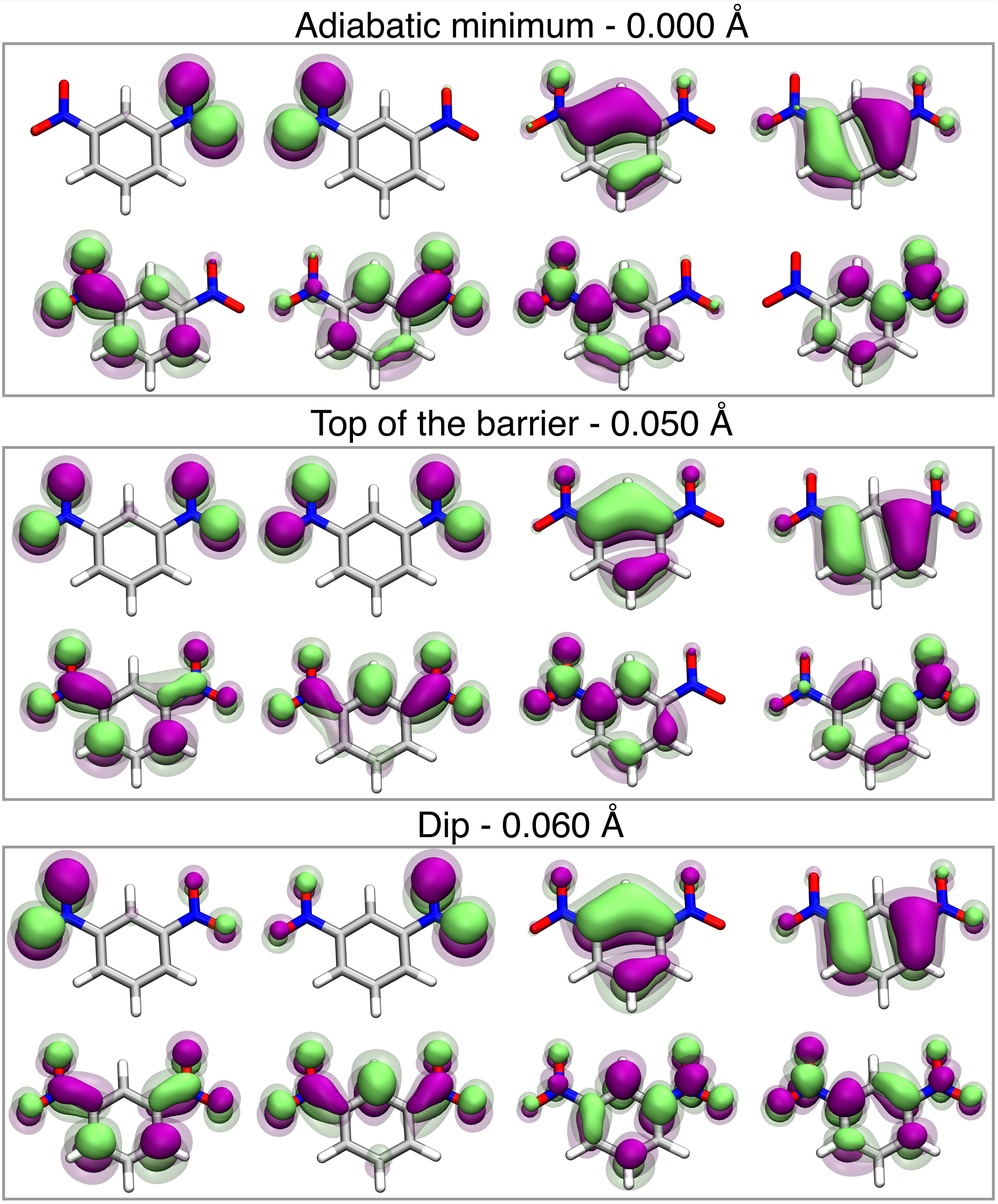

Figure 7: Active space orbitals for the SA-CASSCF/NEVPT2 calculations. The orbitals are shown for three positions in the scan in Fig. 5.

6 Scans along the Marcus dimension (acetonitrile)

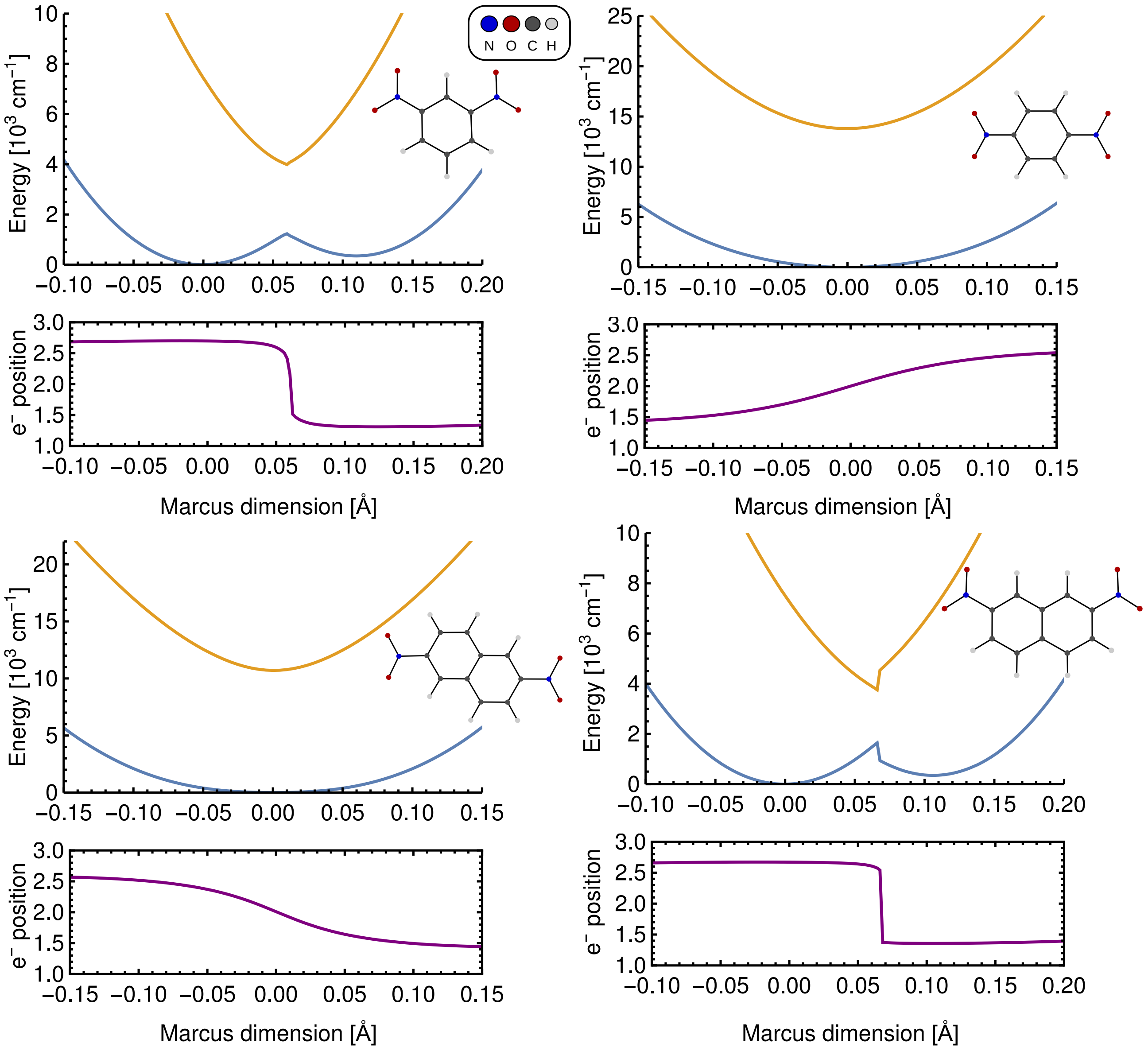

Figure 8: Reconstructed potential energy curves from geometries displaced in the direction of the Marcus dimension for all systems in acetonitrile. For m-DNB$^{\bullet-}$ and 2,7-DNN$^{\bullet-}$, the scan was performed with a step size of 0.002 Å for p-DNB$^{\bullet-}$ and 2,6-DNN$^{\bullet-}$ the step size was 0.005 Å. The change of the electron position is depicted in the bottom panels. The kink in the bottom right panel could not be removed by reconverging the electronic structure.

7 Properties of the Marcus dimension

In practice, the Marcus dimension is described as a normalized $3N$-vector of Cartesian displacements $\mathbf{q}$. The mass of the fictitious particle moving along the Marcus dimension was obtained by weighting the atomic masses with the squared values of their displacement coefficients:

$$\mu = \sum_{i=1}^{3N} q_i^2 m_i. \tag{2}$$

In order to estimate the classical ET rate, a frequency along the Marcus dimension is needed. To obtain this frequency, we can make a use of the fact that the mass of the phonon in the Marcus dimension and the Hessian matrix at the adiabatic minimum are known.

As a first step, the harmonic force constant for the Marcus dimension needs to be computed. To this end, we are going to need the following $3N \times 3N$ matrices: $\mathbf{\Omega}$ as the diagonal matrix of harmonic frequencies, $\mathbf{M}$ as the diagonal matrix of atomic masses, and $\mathbf{C}$ as the matrix of eigenvectors of the Hessian (normal modes) collected as columns. We express the Marcus dimension as a combination of normal coordinates, $\mathbf{a} = \mathbf{C}^T \mathbf{M}^{\frac{1}{2}} \mathbf{q}$, which should be subsequently normalized. The force constant will be computed in a similar manner as the mass in Eq. 2, *i.e.*, combining force constants of normal modes. The harmonic frequency can then be computed as $\omega = \sqrt{\frac{k}{\mu}}$. Combining all of the above results in the following equation for the frequency along the Marcus dimension:

$$\omega = \sqrt{\frac{\mathbf{a}^T \mathbf{\Omega}^2 \mathbf{C}^T \mathbf{M} \mathbf{C} \mathbf{a}}{\mathbf{q}^T \mathbf{M} \mathbf{q}}}. \tag{3}$$

Table 1: Phonon masses and harmonic frequencies for the Marcus dimensions in ACN.

	m-DNB$^{\bullet-}$	p-DNB$^{\bullet-}$	2,6-DNN$^{\bullet-}$	2,7-DNN$^{\bullet-}$
μ [m_u]	14.24801	14.69026	14.47395	14.17365
ω [cm^{-1}]	1007.14	944.48	934.77	1079.69

8 Parameterization of the Marcus model

In the main text of the paper, we described two possibilities for the parameterization of the Marcus model from the *ab initio* scan. The procedure is described in more detail below.

We work with the two diabatic surfaces $G_\mathrm{a} = fx^2$ and $G_\mathrm{b} = f(x-d)^2$. Here, x denotes the progress along the ET coordinate, f the force constant and d the separation of the diabatic states. Under the action of potential coupling V_ab, we obtain the two adiabatic states:

$$G_1 = \frac{1}{2}\left[G_\mathrm{a} + G_\mathrm{b} - \sqrt{\left(G_\mathrm{b} - G_\mathrm{a}\right)^2 + 4V_\mathrm{ab}}\right], \tag{4a}$$

$$G_2 = \frac{1}{2}\left[G_\mathrm{a} + G_\mathrm{b} + \sqrt{\left(G_\mathrm{b} - G_\mathrm{a}\right)^2 + 4V_\mathrm{ab}}\right]. \tag{4b}$$

The properties of the adiabatic states and their spectroscopic implications are already discussed elsewhere in the literature.[15,16] Here, we make use of some important properties described in the following. The excitation energy at the adiabatic minimum, also know as the reorganization energy, is $\lambda = fd^2$. If the potential coupling $2V_\mathrm{ab} < \lambda$, we will obtain a Class II system with a double well potential in the ground state. The position of the barrier will be $d/2$ and the positions of the adiabatic minima $\frac{1}{2}\left(\frac{d^2f \pm \sqrt{d^4f^2 - 4V_\mathrm{ab}^2}}{df}\right)$. The excitation energy at the top of the barrier, *i.e.* position $d/2$, is $2V_\mathrm{ab}$. The barrier height is $\frac{(d^2f - 2V_\mathrm{ab})^2}{4d^2f}$.

From the *ab initio* scan we can extract the following quantities: the excitation energy at the adiabatic minimum $E^\mathrm{Ex.}_\mathrm{min.}$, the excitation energy at the top of the barrier $E^\mathrm{Ex.}_\mathrm{barr.}$, the distance between the adiabatic minimum and the top of the barrier R, and the barrier height ΔG. In the main text, we showed the two possibilities (**A** and **B**) for relating the obtained quantities to the Marcus model.

In parameterization **A**, we expressed the quantities $E^\mathrm{Ex.}_\mathrm{barr.}$, R and ΔG using the adiabatic states G_1 and G_2 from the Marcus model, resulting in three equations:

$$E^\mathrm{Ex.}_\mathrm{barr.} = 2V_\mathrm{ab}, \tag{5a}$$

$$R = d/2 - \frac{1}{2}\left(\frac{d^2f - \sqrt{d^4f^2 - 4V_\mathrm{ab}^2}}{df}\right), \tag{5b}$$

$$\Delta G = \frac{(d^2f - 2V_\mathrm{ab})^2}{4d^2f}. \tag{5c}$$

In parameterization **B**, we expressed the quantities $E^\mathrm{Ex.}_\mathrm{barr.}$, $E^\mathrm{Ex.}_\mathrm{min.}$ and R using the adiabatic states G_1 and G_2 from the Marcus model, again resulting in three equations:

$$E^\mathrm{Ex.}_\mathrm{barr.} = 2V_\mathrm{ab}, \tag{6a}$$

$$R = d/2 - \frac{1}{2}\left(\frac{d^2f - \sqrt{d^4f^2 - 4V_\mathrm{ab}^2}}{df}\right), \tag{6b}$$

$$E^\mathrm{Ex.}_\mathrm{min.} = d^2f. \tag{6c}$$

Either set of Eqs. 5 or 6 can then be solved for f, d and V_ab which are the parameters of a symmetrical Marcus model. The solution can be obtained numerically or using any algebraic software of choice.

9 Heavy atom tunneling

To investigate the relevance of heavy atom tunneling, we computed the transmission coefficient using the semi-classical WKB (Wentzel–Kramers–Brillouin) approximation. The probability of tunneling through the barrier depends on the classical action accumulated between the so-called turning points:[17]

$$T = \exp\left(-\frac{2}{\hbar}\int_{x_1}^{x_2} -i\left[2\mu(E - V(x))\right]^{1/2}\mathrm{d}x\right) \tag{7}$$

Here, T is the transmission coefficient, μ is the mass of the particle, $V(x)$ is the potential energy, E is the total energy of the incident particle and $x_{1,2}$ are the turning points defined as solutions of equation $E = V(x)$.

In our approach, we assume a non-stationary behaviour in the double-well potential, *i.e.*, a particle with energy equal to the harmonic zero point energy (ZPE) is scattered by the potential barrier, see Fig. 9. The turning points $x_{1,2}$ are obtained as solutions of ZPE $= V(x)$. Note that the region $x_1 < x < x_2$ is classically forbidden ($[2\mu(E - V(x))]^{1/2}$ is imaginary). As a potential $V(x)$, the analytical potential from parameterization **A**, presented in the previous section, was used. The ZPE was then calculated as: ZPE $= \frac{1}{2}\hbar(V''(x)|_{x=0}/\mu)^{1/2}$, where μ is the phonon mass from Table 1. The resulting transmission coefficient are presented in Table 2.

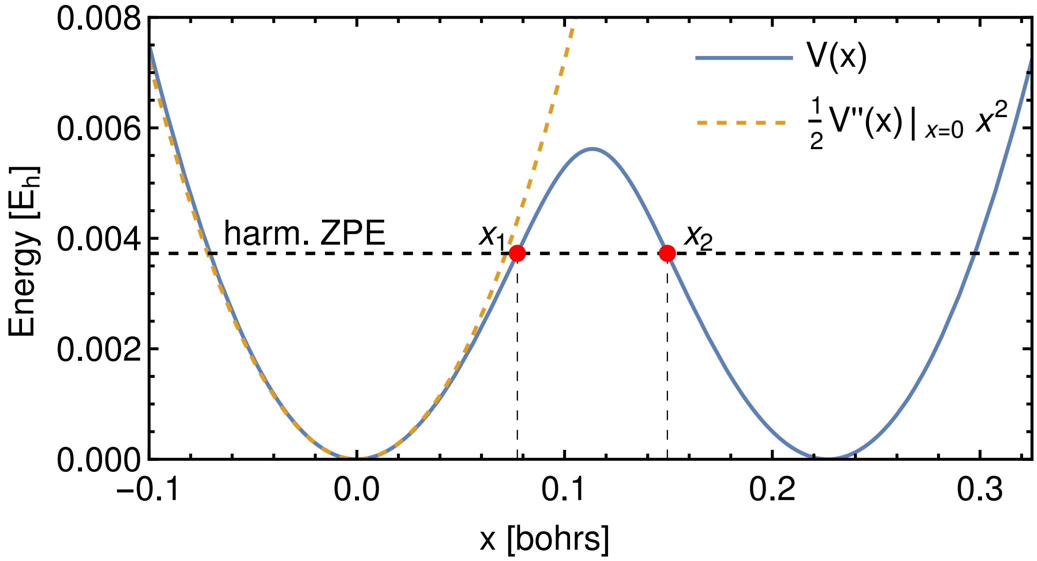

Figure 9: Semi-classical estimation of nuclear tunneling through the potential barrier. $V(x)$ represents the parameterized Marcus model. The energy of the incident particle is taken from harmonic ZPE at the adiabatic minimum (dashed horizontal line). Turning points $x_{1,2}$ represent the boundaries of the classically forbidden region.

Table 2: Semi-classically estimated transmission coefficients for heavy-atom tunneling in the parameterized Marcus model computed according to Eq. 7.

	T
m-DNB$^{\bullet-}$	0.34
2,7-DNN$^{\bullet-}$	0.13

10 Composition of adiabatic states from Marcus–Hush theory

The purpose of this section is to provide an explanation why the Marcus dimension of a class II systems is difficult to obtain by fitting to the electron position. We start with the Marcus–Hush theory, the diabatic states a and b are separated on dimensionless coordinate by 1. Their energies are:

$$G_\mathrm{a} = \lambda X^2, \tag{8a}$$

$$G_\mathrm{b} = \lambda (X-1)^2. \tag{8b}$$

The coupling of the diabatic states V_ab will lead to the adiabatic wavefunctions ψ_1 and ψ_2. The unnormalized coefficients c_a and c_b of the ground state (ψ_1) can be written as:

$$\psi_1 = c_\mathrm{a}\psi_\mathrm{a} + c_\mathrm{b}\psi_\mathrm{b}, \tag{9a}$$

$$\psi_1 = \frac{G_\mathrm{a} - G_\mathrm{b} - \sqrt{(G_\mathrm{a} - G_\mathrm{b})^2 + 4V_\mathrm{ab}^2}}{2V_\mathrm{ab}}\psi_\mathrm{a} + \psi_\mathrm{b}. \tag{9b}$$

It is clear from Eqs. 9 that the admixture of the diabatic state in the adiabatic ground state depends on the progression along the ET coordinate X but also non-trivially on the coupling element V_ab. To elucidate more this dependence the squared absolute values of the normalized coefficients c_a and c_b are plotted in Fig. 10 as a function of X for two different values of V_ab.

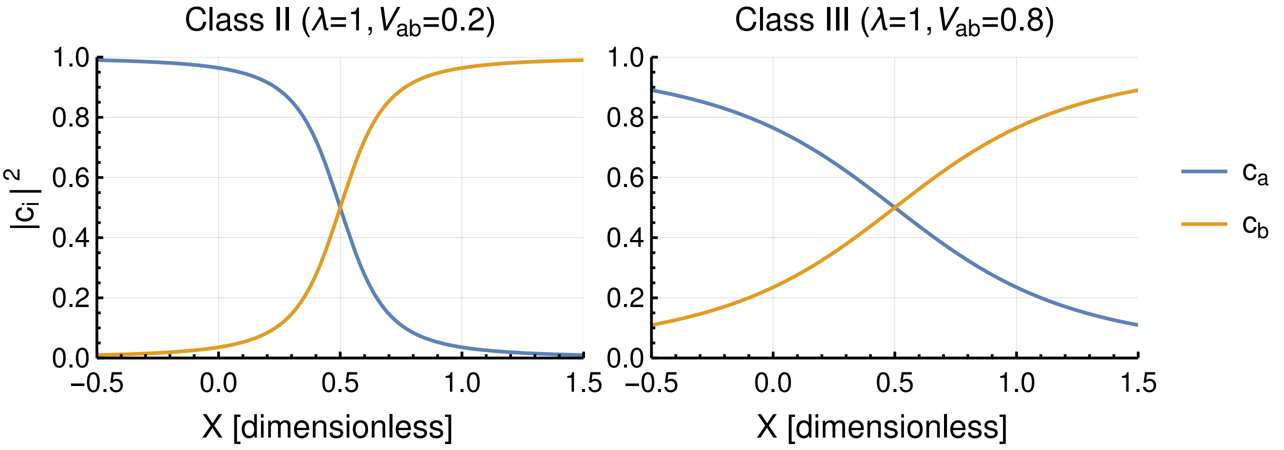

Figure 10: Square absolute value of the diabatic state coefficient in the adiabatic ground state as a function of dimension less coordinate. Left panel depicts a class II situation where $\lambda > 2V_\mathrm{ab}$, right panel depicts a class III situation where $\lambda \leq 2V_\mathrm{ab}$.

In both class II and III situation the dependence of $c_\mathrm{a,b}$ has a sigmoid profile. In class II this profile is sharper (Fig. 10 left), a sampling around adiabatic minimum ($X \approx 0.05$) will result only in a marginal change of the coefficients $c_\mathrm{a,b}$. Hence, the electron density distribution (or the electron position) of the mixed-valent molecule should stay pretty much the same. This will result in a very low variance of electron position in the ensemble of sampled geometries and fitting procedure would be numerically inaccurate. In contrast, sampling around adiabatic minimum of class III system ($X = 0.5$) will provide a sufficiently large variance of the electron position because the admixture of the diabatic state is changing rapidly with X (Fig. 10 right).

11 Cartesian coordinates of the adiabatic minima and Marcus dimensions in acetonitrile

```
16
Adiabatic minimum of m-DNB
N     0.0534117    0.0818807   -0.0037772
O    -0.4093432    0.2538149    1.1085893
O     1.2460100   -0.0254786   -0.2187476
C    -0.8766388    0.0015729   -1.1335960
C    -2.2443505    0.0072879   -0.8711232
C    -3.1023353   -0.0689104   -1.9632628
C    -2.6194300   -0.1422429   -3.2564708
C    -1.2291570   -0.1436742   -3.4906046
C    -0.3446283   -0.0729910   -2.4043909
H    -2.6194359    0.0626883    0.1366040
H    -4.1697574   -0.0714453   -1.7963268
H    -3.2930965   -0.1988802   -4.0956635
N    -0.7309383   -0.2138277   -4.7813634
H     0.7179156   -0.0719201   -2.5737371
O     0.5330778   -0.2153237   -4.9755834
O    -1.5469898   -0.2766557   -5.7639300
```

```
16
Marcus dimension as displacements from equilibrium geometry of m-DNB
N     0.21741     0.02189     0.25727
O     0.08368    -0.03074    -0.20969
O    -0.20205     0.01531     0.04638
C    -0.12359    -0.00310    -0.18371
C     0.01239    -0.00409     0.05452
C     0.00137    -0.00703    -0.03292
C    -0.02656     0.00997     0.06020
C     0.05896    -0.01071    -0.13481
C    -0.01188     0.00515     0.09044
H     0.00640    -0.00125    -0.00084
H    -0.02442     0.00620     0.01646
H     0.02443    -0.00164     0.00872
N    -0.08464     0.02028     0.22440
H    -0.02223    -0.00543    -0.03505
O     0.19088    -0.00349    -0.02791
O    -0.12070    -0.01050    -0.11997
```

```
16
Adiabatic minimum of p-DNB
C     1.3883287    0.0000014   -0.0001248
C    -1.3883306    0.0000022   -0.0001629
C     0.6844576    1.2206751   -0.0001849
C     0.6844567   -1.2206726   -0.0002266
C    -0.6844598   -1.2206673   -0.0002228
C    -0.6844594    1.2206722   -0.0002580
H     1.2247237    2.1534043   -0.0001454
H     1.2247206   -2.1534030   -0.0001192
H    -1.2247152   -2.1534057   -0.0002098
H    -1.2247147    2.1534110   -0.0002622
N    -2.7860736   -0.0000025    0.0001888
O    -3.3993862    1.0870566    0.0000342
O    -3.3993787   -1.0870654    0.0007903
N     2.7860694   -0.0000006    0.0001850
O     3.3993835    1.0870598    0.0008102
O     3.3993781   -1.0870657   -0.0000918
```

```
16
Marcus dimension as displacements from equilibrium geometry of p-DNB
C     0.14212     0.00416     0.01600
C     0.11619     0.01423     0.00683
C    -0.02829     0.02894    -0.01034
C    -0.02365    -0.03729    -0.01187
C    -0.03793     0.01702     0.00311
C    -0.01198    -0.01967     0.00410
H    -0.01190    -0.01059     0.00959
H    -0.00343     0.02970     0.02576
H     0.00990    -0.01267    -0.01944
H     0.00258     0.01175    -0.01408
N    -0.32066    -0.01119     0.00137
O     0.11386    -0.20206    -0.00287
O     0.10929     0.20391    -0.00030
N    -0.32031     0.01566    -0.01216
O     0.10239     0.20393     0.00436
O     0.11833    -0.21638     0.00226
```

```
22
Adiabatic minimum of 2,7-DNN
O    -4.7363499   -0.0567275   -0.0003268
N    -3.6393754   -0.7134188    0.0000416
O    -3.6478774   -1.9910459    0.0005259
C    -2.4286025   -0.0431441   -0.0000839
C    -2.4153084    1.3816805   -0.0000289
C    -1.2362973    2.0623034   -0.0000741
C     0.0086119    1.3794622   -0.0001435
C     1.2380632    2.0671019   -0.0000765
C     2.4325613    1.3946848    0.0000033
C     2.3985110   -0.0113649   -0.0001086
N     3.6633017   -0.7400843    0.0001043
O     4.6983539   -0.0990607    0.0004373
O     3.6356255   -1.9574283   -0.0001001
C     1.2320216   -0.7311429   -0.0002290
C    -0.0053994   -0.0475632   -0.0001452
C    -1.2271958   -0.7453541   -0.0000730
H    -3.3559133    1.9091932    0.0002817
H    -1.2371331    3.1437594    0.0001209
H     1.2310482    3.1483506   -0.0000669
H     3.3721674    1.9218500    0.0001025
H     1.2517087   -1.8099031   -0.0003249
H    -1.2325169   -1.8240210    0.0001219
```

```
22
Marcus dimension as displacements from equilibrium geometry of 2,7-DNN
O    -0.15877     0.10238     0.00019
N     0.19786     0.12987    -0.00352
O     0.01153    -0.18072     0.00077
C    -0.09564    -0.08006     0.00530
C     0.05530     0.01694    -0.00238
C    -0.03721     0.02028    -0.00527
C     0.06089    -0.03979     0.01579
C    -0.03103    -0.00333    -0.01504
C     0.03799    -0.02393     0.00493
C    -0.19184     0.06619    -0.00032
N     0.25336    -0.13495     0.00112
O    -0.14899    -0.08667     0.00270
O    -0.00082     0.17134    -0.00157
C     0.08108     0.01073    -0.01727
C    -0.03755     0.02065     0.01221
C     0.02652     0.00808    -0.00038
H    -0.01180    -0.00616     0.00135
H     0.00256    -0.01535     0.00341
H     0.02825    -0.00490    -0.00047
H     0.00376     0.02145     0.01075
H    -0.00443     0.01974     0.01900
H    -0.00651     0.00592    -0.00480
```

```
22
Adiabatic minimum of 2,6-DNN
C    -1.2546497    2.0797717    0.0001235
C    -0.0000631    1.3977050    0.0001338
C    -0.0072417   -0.0377413   -0.0000480
C    -1.2151265   -0.7254839    0.0000216
C    -2.4188163   -0.0234128    0.0000229
C    -2.4294719    1.3978343    0.0001257
C     1.2473871   -0.7196630    0.0000150
C     2.4223228   -0.0376175    0.0000986
C     2.4109720    1.3832319    0.0000806
C     1.2078058    2.0854197    0.0000932
H     3.3647859   -0.5613149    0.0003642
N     3.6244954    2.0845518   -0.0002001
O     4.6979451    1.4520933   -0.0012522
O     3.6091955    3.3306896    0.0006455
N    -3.6314432   -0.7236435   -0.0001752
O    -3.6169328   -1.9709105    0.0005523
O    -4.7060382   -0.0908501   -0.0011098
H    -3.3719541    1.9214969    0.0003369
H    -1.2563731    3.1609716    0.0000347
H     1.2151217    3.1645934   -0.0001740
H     1.2492774   -1.8008554    0.0001141
H    -1.2224745   -1.8046403    0.0000668
```

```
22
Marcus dimension as displacements from equilibrium geometry of 2,6-DNN
C    -0.00075    -0.00711    -0.00639
C    -0.00342     0.01134    -0.00012
C     0.02773     0.00966    -0.01685
C     0.02201     0.00064     0.01187
C    -0.08090    -0.05271     0.00205
C     0.01276     0.01545     0.00064
C    -0.00531    -0.00442     0.00961
C    -0.00116     0.02085    -0.00940
C    -0.08318    -0.06370    -0.00097
C     0.01217    -0.02135     0.01184
H    -0.00252    -0.02696     0.01170
N     0.21885     0.11422    -0.00005
O    -0.15139     0.08351     0.00056
O     0.00302    -0.14455    -0.00005
N     0.20938     0.10311    -0.00395
O     0.00335    -0.15450    -0.00014
O    -0.15395     0.09304     0.00208
H     0.00791    -0.01913    -0.01135
H     0.00694     0.00160     0.00936
H    -0.00690     0.03658    -0.00991
H     0.00118     0.01287    -0.00458
H    -0.01930     0.00802    -0.00581
```

References

1. Launay, J.-P. & Verdaguer, M. *Electrons in Molecules* 283 (Oxford University Press, 2018).
2. Haasler, M., Maier, T. M., Grotjahn, R., Gückel, S., Arbuznikov, A. V. & Kaupp, M. A Local Hybrid Functional with Wide Applicability and Good Balance between (De)Localization and Left–Right Correlation. *Journal of Chemical Theory and Computation* **16,** 5645–5657 (2020).
3. Renz, M., Theilacker, K., Lambert, C. & Kaupp, M. A reliable quantum-chemical protocol for the characterization of organic mixed-valence compounds. *Journal of the American Chemical Society* **131,** 16292–16302 (2009).
4. Becke, A. D. Density-functional thermochemistry. IV. A new dynamical correlation functional and implications for exact-exchange mixing. *The Journal of Chemical Physics* **104,** 1040–1046 (1996).
5. Neese, F. Software update: The ORCA program system–Version 5.0. *WIREs Computational Molecular Science* **12,** 1–15 (2022).
6. Weigend, F. & Ahlrichs, R. Balanced basis sets of split valence, triple zeta valence and quadruple zeta valence quality for H to Rn: Design and assessment of accuracy. *Physical Chemistry Chemical Physics* **7,** 3297–3305 (2005).
7. Stoychev, G. L., Auer, A. A. & Neese, F. Automatic Generation of Auxiliary Basis Sets. *Journal of Chemical Theory and Computation* **13,** 554–562 (2017).
8. Telo, J. P., Jalilov, A. S. & Nelsen, S. F. Effect of ortho substitution on the charge localization of dinitrobenzene radical anions. *Journal of Physical Chemistry A* **115,** 3016–3021 (2011).
9. Nelsen, S. F., Weaver, M. N., Konradsson, A. E., Telo, J. P. & Clark, T. Electron transfer within 2,7-dinitronaphthalene radical anion. *Journal of the American Chemical Society* **126,** 15431–15438 (2004).
10. Koch, A., Kinzel, D., Dröge, F., Gräfe, S. & Kupfer, S. Photochemistry and Electron Transfer Kinetics in a Photocatalyst Model Assessed by Marcus Theory and Quantum Dynamics. *The Journal of Physical Chemistry C* **121,** 16066–16078 (2017).
11. Parthey, M., Gluyas, J. B. G., Fox, M. A., Low, P. J. & Kaupp, M. Mixed-Valence Ruthenium Complexes Rotating through a Conformational Robin–Day Continuum. *Chemistry - A European Journal* **20,** 6895–6908 (2014).
12. Kollmar, C., Sivalingam, K., Helmich-Paris, B., Angeli, C. & Neese, F. A perturbation-based super-CI approach for the orbital optimization of a CASSCF wave function. *Journal of Computational Chemistry* **40,** 1463–1470 (2019).
13. Angeli, C., Cimiraglia, R. & Malrieu, J.-P. N-electron valence state perturbation theory: a fast implementation of the strongly contracted variant. *Chemical Physics Letters* **350,** 297–305 (2001).
14. Kaupp, M., Karton, A. & Bischoff, F. A. $[Al_2O_4]^-$, a Benchmark Gas-Phase Class II Mixed-Valence Radical Anion for the Evaluation of Quantum-Chemical Methods. *Journal of Chemical Theory and Computation* **12,** 3796–3806 (8 2016).
15. Brunschwig, B. S., Creutz, C. & Sutin, N. Optical transitions of symmetrical mixed-valence systems in the class II-III transition regime. *Chemical Society Reviews* **31,** 168–184 (2002).
16. Heckmann, A. & Lambert, C. Organic mixed-valence compounds: A playground for electrons and holes. *Angewandte Chemie - International Edition* **51,** 326–392 (2012).
17. Schatz, G. C. & Ratner, M. A. *Quantum Mechanics in Chemistry* 1., 167–172 (Dover Publications, New York, 2002).
